# Association between dietary magnesium intake, inflammation, and neurodegeneration

**DOI:** 10.1007/s00394-024-03383-1

**Published:** 2024-04-10

**Authors:** Khawlah Alateeq, Erin I. Walsh, Ananthan Ambikairajah, Nicolas Cherbuin

**Affiliations:** 1 National Centre for Epidemiology and Population Health, Australian National University, 54 Mills Road, Canberra, ACT 2601 Australia; 2 Radiological Science, College of Applied Medical Science, King Saud University, Riyadh, Saudi Arabia; 3 Discipline of Psychology, Faculty of Health, University of Canberra, Canberra, ACT 2617 Australia; 4 Centre for Ageing Research and Translation, Faculty of Health, University of Canberra, Canberra, 2617 Australia

**Keywords:** Magnesium, Inflammation, Brain volumes, White matter lesions, UK biobank

## Abstract

**Background:**

Consistent evidence shows that magnesium (Mg) intake is associated with lower blood pressure (BP), and that lower BP is associated with improved cerebral health. However, recent findings indicate that the positive effect of dietary Mg intake on cerebral health is not mediated by a decrease in BP. As Mg’s anti-inflammatory action is a plausible alternative mechanism, the objective of this study was to investigate the associations between Mg intake and inflammation to determine whether it mediates any neuroprotective effect.

**Methods:**

Participants from the UK Biobank (*n* = 5775, aged 40–73 years, 54.7% female) were assessed for dietary magnesium using an online food questionnaire, brain and white matter lesion (WML) volumes were segmented with FreeSurfer software, and inflammation markers including high-sensitivity C-reactive protein (hs-CRP), leukocyte, erythrocyte count, and Glycoprotein acetylation (GlycA) were measured using specific laboratory techniques such as immunoturbidimetry, automated cell counting, and nuclear magnetic resonance. Hierarchical linear regression models were performed to investigate the association between dietary Mg, and inflammatory markers and between dietary Mg, brain and WMLs volumes. Mediation analysis was performed to test a possible mediation role of inflammation on the association between dietary Mg and brain and WMLs volumes.

**Results:**

Higher dietary Mg intake was associated with lower inflammation: hs-CRP level (− 0.0497%; 95% confidence interval [CI] − 0.0497%,  − 0.0199%) leukocytes count (− 0.0015%; 95%CI − 0.00151%,  − 0.0011%), and GlycA (− 0.0519%; 95%CI − 0.1298%,  − 0.0129%). Moreover, higher dietary Mg intake was associated with larger grey matter volume (0.010%; 95%CI 0.004%, 0.017%), white matter volume (0.012%; 95%CI 0.003,  0.022) and right hippocampal volume (0.002%; 95%CI 0.0007, –0.0025%). Lower hs-CRP levels mediated the positive association between higher dietary Mg intake and larger grey matter volume.

**Conclusions:**

The anti-inflammatory effects of dietary Mg intake in the general population, appears to mediate its neuroprotective effect.

**Supplementary Information:**

The online version contains supplementary material available at 10.1007/s00394-024-03383-1.

## Introduction

Ageing of the population is projected to lead to an increase in the proportion of individuals aged 65 years and older, with estimates suggesting it will increase from 10% today to 16% of the world’s population by 2050 [1]. This trend has important healthcare and economic implications for our society [2]. In particular, older age is associated with an increase prevalence of dementia and other neurodegenerative disease. Therefore, there is a pressing need for the development of effective strategies to promote healthy brain ageing and mitigate the risk of neurodegenerative diseases and dementia, both for the benefit of individuals and for the sustainability of healthcare systems.

Nutrition is an important modifiable risk factor that influences cerebral health and that is highly amenable to interventions that are scalable and cost-effective [3]. Dietary magnesium (Mg), in particular, is associated with better cognitive function [4] and may reduce the risk and delay the onset of dementia [5]. However, the underlying biological mechanisms responsible for the neuroprotective effect of Mg are not well understood. This is a crucial question to address, as optimising Mg intake through diet may contribute to reducing the risk of dementia in the general population.

Mg plays an important role in neuronal health. It is essential for nerve transmission and neuromuscular conduction [6]. Mg deficiency has been linked to the development of several neurological pathologies related to ageing [7]. A recent study that investigated the link between Mg and brain volumes in a large population (*n* = 6000; mean age 40–70 years) found that higher dietary Mg intake was associated with larger brain volumes, particularly in the hippocampus [8]. Another study (*n* = 1406; mean age = 62.5 years) with an eight-year follow-up found that higher dietary Mg intake (≥ 434 mg; recommended minimum intake 350-400 mg) was also linked to a lower risk of progressing from normal cognition to mild cognitive impairment (MCI; hazard ratios [HR] = 0.07, 95% confidence interval [CI] 0.01, 0.56) [9]. In a recent study with 1565 participants (mean age 71.7 years) from an urban Shanghai community, it was found that high Mg intake (> 267.5 mg/day) at baseline was linked to an elevated risk of dementia within a 5-year follow-up period [10]. It is important to note that the study had a relatively short follow-up duration, and despite dementia cases were excluded, participants with MCI were not excluded in this study [10]. Moreover, a recent systematic review demonstrated that individuals with Alzheimer’s disease (AD) have significantly lower plasma Mg levels (standardized mean difference [SMD] = − 0.89; 95%CI − 1.36, − 0.43) than healthy controls [11]. Contrarily, a study involving 102,648 individuals showed no association between plasma Mg concentrations and AD, suggesting a reverse causation of explanation [12]. It is also possible that these contradictory results are due to the fact that Mg is primarily stored intracellular, with only around 1% found in the bloodstream. As a result, serum Mg levels may not accurately represent total Mg levels in the body, underscoring the importance of dietary intake in assessing Mg levels [13]. In combination, these findings suggest that dietary Mg intake contributes to the modulation of neurodegenerative processes, and further research is needed to better understand this relationship.

However, the precise mechanisms underlying Mg’s neuroprotective effects remain unclear. A plausible mechanism is the known anti-hypertensive effect of Mg, as numerous studies have indicated that Mg supplementation lowers blood pressure (BP) and helps in the management of hypertension [14, 15], because Mg acts as a calcium antagonist on the smooth muscles, leading to vasorelaxation [16]. Since elevated BP is closely linked to neurodegeneration [17–20], cognitive decline [21], and dementia [22], we recently conducted research in a large population to investigate the link between dietary Mg intake and BP and examined whether BP mediates any neuroprotective effects. Contrary to expectations, our findings did not support a blood-lowering effect as the main mechanism mediating the relationship between Mg and cerebral health. This suggests that other mechanisms may be involved [8].

A potential alternative mechanism is the anti-inflammatory effect of Mg. Animal studies have shown that low Mg intake is associated with microglia activation and the production of pro-inflammatory cytokines, including interleukin 1 beta (IL-1), IL-6, and tumour necrosis factor alpha (TNF-α) [23]. Mg supplementation, in contrast, has an anti-inflammatory impact by reducing the production of pro-inflammatory cytokines, including IL-1, IL-6, and TNF-α [24]. Moreover, a recent systematic review including 11 randomized controlled trials (RCTs) found that Mg supplementation significantly decreased C-reactive protein (CRP) serum levels (SMD = − 0.356; 95%CI − 0.659,−0.054) [25]. CRP is produced by the liver in response to the acute inflammatory phase following infections or trauma, and its production is regulated by IL-1 and IL-6 [26]. Moreover, it is worth noting that there is evidence suggesting a link between higher inflammation markers including IL-6 and CRP levels and an increased risk of neurodegeneration [27], cognitive impairment [28], and dementia [29, 30]. However, conflicting evidence suggests no discernible differences in CRP levels between AD patients and controls [31].

Taken together, the current evidence suggests that dietary Mg intake has a positive impact on reducing inflammation and protecting cerebral health. However, it is unknown whether its anti-inflammatory action is responsible for its neuroprotective effect. Consequently, the aim of this study was to investigate the association between dietary Mg and high-sensitivity CRP (hs-CRP), an indicator of inflammation. Due to the unavailability of Interleukins such as, IL-1 and IL-6 in the UK Biobank database, the study also aimed to explore the associations between Mg intake and other available inflammatory markers, including leukocyte and erythrocyte count, and Glycoprotein acetylation (GlycA) levels. The leukocyte count, in particular, is important as it is a key component of the innate immune system’s defence mechanism and is responsible for expressing and secreting Interleukins [32, 33] Recent studies also suggest that erythrocytes are vulnerable to oxidative stress and upregulation of cytokines which are associated with inflammatory diseases [34]. Furthermore, the GlycA biomarker has been used to assess pro-inflammatory cytokines and predicts the development of cardiovascular disease [35–38]. Finally, the second aim of this study was to examine whether any effect of Mg on inflammation mediates the associations between Mg and brain and WMLs volumes, as measures of cerebral health.

## Methods

### Study design and participants

Participants recruited into the UK biobank study, which has previously been described [39], were considered for inclusion in this study. Briefly, the UK Biobank is a prospective cohort study of 502,655 participants aged 37 to 73 years at baseline who were evaluated at 22 assessment centres across the United Kingdom between 2006 and 2023. Participants who had both baseline diastolic BP (DBP) and systolic BP (SBP) measurements (*n* = 456,990) at baseline in 2006–2009, completed a structural magnetic resonance imaging (MRI) scan at the second assessment in 2014 (*n* = 36,260), for whom dietary Mg intake and inflammatory markers at baseline in 2006–2009 (*n* = 30,484), and who did not have any neurological disordered (*n* = 3,275) were excluded. This resulted in a final sample of participants (*n* = 5776 leukocytes and erythrocytes, *n* = 5641 hs-CRP, and *n* = 1457 GlycA; Supplementary material; Fig. S1).

The North-West Multi-Centre Research Ethics Committee approved the UK Biobank Study (#06/MRE08/65). All participants provided informed consent. This study follows to the STROBE (Strengthening the Reporting of Observational Studies in Epidemiology) guidelines [40].

### Measurement of mg intake Dietary

Dietary intake was measured using the Oxford WebQ, a computerised 24-hour recall questionnaire administered online [41, 42]. The WebQ was designed to be completed multiple times to minimize measurement error that might occur with a single 24-hour recall assessment. It includes 200 items in various quantity sizes. The overall dietary Mg was computed using McCance and Widdowson’s “The Composition of Food and its Supplements” [42]. Specific details regarding the calculation of Mg intake are described elsewhere [41, 42].The WebQ has been validated against a 24-hour recall assessment performed by an interviewer, with only minor discrepancies in nutrient intake reported using both procedures [42].

### MRI acquisition

MRI images were collected at one of three imaging locations using the same scanner (3T Siemens Skyra, running VD13A SP4 using a 32-channel head coil). Detailed imaging protocol are available online (http://biobank.ctsu.ox.ac.uk/crystal/refer.cgi?id=1977) [39]. Briefly, T1-weighted brain MRI scans were acquired in sagittal orientation using a 3D magnetization-prepared rapid acquisition gradient echo sequence (resolution = 1 × 1 × 1 mm; matrix size = 208 × 256 × 256; T1/TR = 880/2000 ms).

### Segmentation and image analysis

MRI data was segmented and analysed using FreeSurfer (version6.0.5) [43]. The FreeSurfer pipeline has been detailed elsewhere [44], but in brief, it includes motion correction, transformation to Talairach image space, inhomogeneity normalisation, non-brain tissue removal using hybrid watershed, volumetric segmentation [45, 46], and cortical surface reconstruction and parcellation [47]. The region of interest (ROI) was selected based on previous investigation showing a relationship between dietary Mg and brain ageing [8]. They included total grey matter volume (GM), total white matter volume (WM), left and right hippocampus volume (LHC, RHC) and white matter lesions (WMLs).

### Inflammatory markers

Inflammatory markers included: hs-CRP level (mg/L) serum was assessed using immunoturbidimetric high-sensitivity analysis on a Beckman Coulter AU5800 [31–33]. leukocytes count (10^9^ cells/Litter) and erythrocyte count (10^12^ cells/Litter) were measured as an absolute number per unit volume on fresh samples using an automated, clinically validated Coulter LH 750 (Beckman Coulter). Calibration and quality control were carried out in accordance with the manufacturer’s instructions. GlycA (mmol/Litter) was measured using an NMR metabolomics platform (Nightingale Health, Helsinki, Finland). More information can be found on the UK Biobank website (http://www.ukbiobank.ac.uk).

### Covariates

The covariates included age, sex, body mass index (BMI), serum high-density lipoprotein cholesterol (HDL-C), total cholesterol (TC), education level, diabetes mellitus diagnosed by a doctor, self-reported smoking status (i.e., never, previous, or current), diagnosed with hypertension (participants with SBP/DBP of ≥ 140/90 or who reported taking BP medication), alcohol intake (drinks/week), and physical activity (metabolic equivalent [METs]/week) [48].

### Statistical analyses

Statistical analyses were computed using the R statistical package (Version 1.2.5019) under Rstudio (Version 1.2.5019) [49]. Descriptive analyses were conducted using Chi-square tests for categorical data and t-tests to compare groups on continuous variables. The skewed distribution of the WMLs was transformed using log transformations. All dependent and independent variables remained unstandardized. Mg intake was centered on 350 mg (recommended daily intake ~ 310–420) to facilitate interpretation [50].

Hierarchical linear regression models were performed to investigate the association between (1) baseline Mg intake and inflammatory markers (hs-CRP, leukocytes, erythrocytes, and GlycA); and (2) baseline Mg intake and brain volumes (GM, WM, LHC, RHC, WMLs). The main three models were fit as follows: Model 1 was controlled for age, sex and education. Model 2 additionally controlled for the cardiovascular risk factors including diabetes mellitus, hypertension, BP medication, HDL, TC, alcohol intake, physical activity, smoking status, BMI and the time difference between baseline and follow-up, owing to significant variability in the time span with average 8 years. Model 3 additionally tested the two-way interactions between baseline Mg intake and the cardiovascular risk factors. Sensitivity analyses were conducted to evaluate the influence of calcium (Ca), considering the biological link between Mg and Ca [51]. Additionally, adjustments for energy intake were performed to address confounding variables related to high energy associated with high food intake. Since a prior study has highlighted sex differences in Mg intake analysis [52], a sensitivity analysis was performed to investigate sex differences in the association between Mg and brain volumes and Mg and inflammation by stratifying the sample into men and women. All brain analyses were controlled for the intra-cranial volume (ICV), to correct for head size differences. The unstandardized beta coefficient, standard error, and p-values for outcomes measures are reported. The significance threshold was set at *p* < 0.05 and corrected for multiple comparisons (Bonferroni).

Possible mediation of dietary Mg intake on the brain volume and WMLs through inflammation was investigated using Baron and Kenny’s method [53, 54]. The bootstrapping of indirect effect was set to *n* = 1000. The three main steps were tested. Step 1 tested the effect of dietary Mg intake and other covariates on the brain volumes and WMLs. Step 2 tested the effects of dietary Mg intake and other covariates on the inflammatory markers meeting criteria. Step 3 tested the total effects of inflammatory markers, dietary Mg, and other covariates on brain volumes and WMLs. Step 4 tested the causal mediation analysis of the indirect effect of inflammation on brain volumes and WMLs through dietary Mg.

## Results

### Participants characteristics

Participant characteristics are presented in Table 1. While the average Mg intake (mean = 361.9, SD = 125.11) was above the recommended 350 mg mg/day, this was the case for men (mean = 383.83, SD = 133.03), but not women (mean = 342.16, SD = 113.91) even though it was within the normal range for both sexes. Moreover, men were slightly older (~ 1year) and had slightly higher BMI (1.2 ${\text{k}\text{g}/\text{m}}^{2}$), SBP (6.5mmHg), DBP (3.84mmHg) and had a higher prevalence of BP medication (1.8%) and diabetes mellitus (1.6%), than women.

**Table 1:** Participants demographic characteristic

Measures	Whole Sample	Males	Females	(P value)
	(*n* = 5766)	(*n* = 2745)	(*n* = 3021)	
Age, year (SD)	55.37 (7.45)	56.05 (7.51)	54.75 (7.35)	(< 0.001)
Magnesium intake, mg	361.99 (125.11)	383.83 (133.03)	342.16 (113.91)	(< 0.001)
GM volume, mm^3^ (SD)	668753.30 (59323.58)	702489.76 (53567.20)	638115.21 (46234.46)	(< 0.001)
WM volume, mm^3^ (SD)	479969.55 (57361.26)	510020.62 (54142.82)	452678.38 (45297.32)	(< 0.001)
Left HC volume, mm^3^ (SD)	3696.18 (395.93)	3829.82 (401.13)	3574.81 (349.42)	(< 0.001)
Right HC volume, mm^3^ (SD)	3811.71 (402.65)	3951.46 (409.86)	3684.79 (350.71)	(< 0.001)
WMLs volume, mm^3^(SD)	7.38 (0.66)	7.50 (0.65)	7.26 (0.64)	(< 0.001)
ICV volume, mm^3^ (SD)	1554471.18 (150902.35)	1643379.11 (134101.86)	1473728.56 (115865.44)	(< 0.001)
Leukocyte count, 10^9^ cells/L (SD)	6.59 (1.78)	6.60 (1.60)	6.58 (1.93)	(0.645)
Erythrocyte count, 10^12^ cells/Litre (SD)	4.55 (0.40)	4.78 (0.35)	4.34 (0.32)	(< 0.001)
hs-CRP Level, mg/L	2.01 (3.32)	1.85 (2.95)	2.15 (3.61)	(< 0.001)
Glycoprotein Acetyls, mmol/L	0.77 (0.11)	0.76 (0.11)	0.77 (0.11)	(0.634)
SBP, mmHg (SD)	134.77 (17.83)	138.20 (16.60)	131.66 (18.32)	(< 0.001)
DBP, mmHg (SD)	81.15 (9.95)	83.17 (9.91)	79.31 (9.63)	(< 0.001)
BMI, kg/m^2^ (SD)	26.51 (4.14)	27.08 (3.77)	26.00 (4.39)	(< 0.001)
Cholesterol, mmol/L, (SD)	5.74 (1.07)	5.62 (1.08)	5.86 (1.06)	(< 0.001)
HDL mmol/L, (SD)	1.49 (0.36)	1.32 (0.29)	1.64 (0.36)	(< 0.001)
Hypertension, n (%)	2352 (40.65%)	1312 (47.81%)	1030 (34.08%)	(< 0.001)
BP medication, n (%)	435 (7.53%)	231 (8.40%)	204 (6.74%)	(0.019)
Diabetes, n (%)	163 (2.82%)	97 (3.53%)	66 (2.18%)	(0.003)
Higher Education, n (%)	2845 (49.26%)	1427 (51.91%)	1418 (46.85%)	(< 0.001)

Significance: *p* < 0.05. Abbreviation; SBP: systolic blood pressure; DBP: diastolic blood pressure; GM: grey matter; WM: white matter; HC: hippocampus; WMLs: white matter lesions; ICV: intracranial volume; BMI: body mass index; HDL: high-density lipoprotein; hs-CRP: high-sensitivity c-reactive protein. Note. The statistical test reported based on the group comparison of men relative to women

### Dietary mg intake and inflammation

Associations between Mg, and inflammation levels are presented in Table 2. Higher dietary Mg intake was significantly associated with lower inflammation levels, with some variation across inflammatory markers. Every additional 1 mg in Mg intake above 350 mg/day was associated with a – 0.049% lower hs-CRP, − 0.0015% lower leukocytes, and − 0.0519% lower GlycA. However, this association did not reach the significance for erythrocyte levels (Table S1-S5 and Fig. S2-S6).

**Table 2:** Association between dietary Mg intake and inflammatory markers

	Leukocytes (10^9^ cells/L)	ESR (10^12^ cells/L)	hs-CRP Level (mg/L)	GlycA (mmol/L)
	Beta (CI)	Beta (CI)	Beta (CI)	Beta (CI)
Mg	-0.0001^*^ (-0.0001, -0.00007) *p* = 0.01925	-0.00001 (-0.0001, 0.0001) *p* = 0.21675	-0.001^***^ (-0.001, -0.0004) *p* = 0.00000	-0.0004^***^ (-0.001, -0.0001) *p* = 0.00075
BP medication	0.010 (-0.014, 0.035) *p* = 0.403	0.006 (-0.028, 0.039) *p* = 0.737	0.457^***^ (0.157, 0.757) *p* = 0.003	0.012 (-0.009, 0.034) *p* = 0.264
Hypertension	0.013^*^ (-0.0002, 0.027) *p* = 0.054	0.073^***^ (0.054, 0.092) *p* = 0.00	-0.087 (-0.244, 0.071) *p* = 0.281	0.006 (-0.005, 0.017) *p* = 0.306
Smoking status	0.044^***^ (0.034, 0.054) *p* = 0.00	-0.043^***^ (-0.057, -0.029) *p* = 0.00	0.079^***^ (0.038, 0.119) *p* = 0.0002	0.010^**^ (0.001, 0.019) *p* = 0.356
Cholesterol	0.001 (-0.005, 0.007) *p* = 0.707	0.041^***^ (0.033, 0.050) *p* = 0.000	0.070^***^ (0.046, 0.094) *p* = 0.00	0.032^***^ (0.027, 0.037) *p* = 0.00
Mg x Smoking status	0.0001^**^ (0.00001, 0.0002) *p* = 0.033	—	—	—
Mg x BP medication	—	—	-0.001^***^ (-0.002, -0.0003) *p* = 0.033	—
Mg x Hypertension	—	—	0.0004^**^ (0.00004, 0.001) *p* = 0.032	—
Mg x Cholesterol	—	—	—	0.0001^***^ (0.00002, 0.0001) *p* = 0.005
Constant	-4.292^***^ (-4.368, -4.217) *p* = 0.000	4.536^***^ (4.432, 4.640) *p* = 0.000	-2.826^***^ (-3.139, -2.513) *p* = 0.000	0.503^***^ (0.441, 0.566) *p* = 0.000
Observations	5,766	5,766	5,468	1,417

Significance. * *p* < 0.05; ** *p* < 0.01; *** *p* < 0.001. Abbreviations: CI-confidence interval - standard error; Mg - magnesium; hs-CRP - high-sensitivity C-reactive protein; GlycA - Glycoprotein acetylation. Note: The hierarchical analysis presents results on the association between dietary Mg intake and inflammatory markers, including leukocytes, erythrocytes, hs-CRP, and GlycA, using data from the UK Biobank study. In Model 3, adjustments were made for covariates including as age, sex, education, BP medication, high-density lipoprotein (HDL), cholesterol, diabetes mellitus, smoking status, higher education, physical activity, alcohol intake and body mass index (BMI). We also tested two-way interactions between Mg and smoking status, Mg and hypertension, Mg and BP medication, and Mg and cholesterol levels. The data represents unstandardized beta coefficients with 95% Confidence Interval (CI). Beta values correspond to a 1 mg unit increment in Mg intake variables

### Effects of cardiovascular risk factors

Two-way interactions between Mg intake and cardiovascular risk factors as predictors of the inflammatory markers are presented in Table 2 and Fig. S7. Cardiovascular risk factors were found to significantly modulate the association between Mg intake and inflammation with some notable differences between markers.

The negative association between Mg intake and hs-CRP was weaker (0.029% lower leukocyte for every 1 mg Mg above 350 mg/day) in hypertensive. However, the negative association between Mg intake and hs-CRP was stronger (–0.059% lower hs-CRP for every 1 mg Mg above 350 mg/day) in those who were treated with BP medication.

Moreover, the negative association between Mg intake and leukocytes was weaker (0.001% lower leukocyte for every 1 mg Mg above 350 mg/day) in those who smoked. Also, association between Mg and GlycA was weaker (0.013% lower GlycA for every 1 mg Mg above 350 mg/day) with higher cholesterol levels.

### Sensitivity analysis

Sensitivity analyses were performed to account for Ca and energy intake when assessing the relationship between dietary Mg intake and inflammatory markers. The significant association between dietary Mg intake and inflammatory markers remained unchanged after adjusting for Ca, suggesting that this association is independent of Ca (Table S12). Furthermore, the association between dietary Mg intake and inflammatory markers persisted even after controlling for energy intake, except for the association between dietary Mg and ESR levels, which became stronger (-0.002%) (Table S12).

Additional analyses stratified by sex were conducted to better characterise sex-specific associations between Mg intake and inflammation levels, (Table S13). Higher dietary Mg intake was significantly associated with lower leucocyte levels in women, with a weaker association in men. Furthermore, higher dietary Mg intake was significantly associated with lower GlycA in men, and this association was weaker in women.

### Dietary mg intake and brain volumes and WMLs

Associations between baseline Mg, and brain and WMLs volumes are presented in Table 3. Higher dietary Mg intake was significantly associated with larger brain volumes. Every 1 mg higher in Mg intake above 350 mg/day was associated with a 0.0105% larger GM, 0.0122% larger WM, and 0.002% larger RHC. The association did not reach significance for LHC, and WMLs (Table S6-S11 and Fig. S8-S13).

**Table 3:** Association between dietary Mg intake and brain volumes and WMLs.

	Gray matter volume (mm^3^)	White matter volume (mm^3^)	Left hippocampal volume (mm^3^)	Right hippocampal volume (mm^3^)	White matter lesions volume (mm^3^)
	Beta (CI)	Beta (CI)	Beta (CI)	Beta (CI)	Beta (CI)
Mg	70.321^***^ (26.028, 114.615) *p* = 0.0004	58.751^**^ (13.766, 103.735) *p* = 0.002	0.021 (-0.043, 0.086) *p* = 0.103	0.063^*^ (0.029,0.095) *p* = 0.012	0.00003 (-0.0001, 0.0001) *p* = 0.123
Age	-1,574.842^***^ (-1,679.220, -1,470.465) *p* = 0.000	-1,100.555^***^ (-1,206.560, -994.549) *p* = 0.000	-16.550^***^ (-17.667, -15.434) *p* = 0.000	-15.325^***^ (-16.455, -14.194) *p* = 0.000	0.041^***^ (0.039, 0.043) *p* = 0.000
Mg x Age	-1.151^***^ (-1.946, -0.356) *p* = 0.005	-1.024^**^ (-1.832, -0.217) *p* = 0.013			
Constant	300,081.900^***^ (285,775.300, 314,388.400) *p* = 0.000	85,425.380^***^ (70,895.690, 99,955.070) *p* = 0.000	2,699.007^***^ (2,545.463, 2,852.551) *p* = 0.000	2,583.895^***^ (2,428.393, 2,739.397) *p* = 0.000	2.601^***^ (2.333, 2.869) *p* = 0.000
Observations	5,766	5,766	5,766	5,766	5,766

Significance. * *p* < 0.05; ** *p* < 0.01; *** *p* < 0.001. Abbreviations: CI - confidence interval; Mg - magnesium; GM - grey matter; WM - white matter; LHC - left hippocampal; RHC - right hippocampal; WMLs - white matter lesions. Note: This section presents the hierarchical analysis results of the association between Mg intake and brain volumes, including GM, WM, LHC, RHC, and WMLs. In Model 3, adjustments were made for covariates including age, sex, education, ICV (intracranial volume), BP medication, high-density lipoprotein (HDL), cholesterol, diabetes mellitus, smoking status, higher education, physical activity, alcohol intake, body mass index (BMI), and time differences between measurements. Additionally, we tested the two-way interaction between Mg and age. The data represents unstandardized beta coefficients with 95% Confidence Interval (CI). Beta values correspond to a 1 mg unit increment in Mg intake variables

### Effects of age

Two-way interactions between Mg and age as predictors of the brain volumes are presented in Table 3 and **Fig. S14.** Age was found to significantly modulate the association between Mg intake and brain volumes. Therefore, a further analysis stratified by age group (age ≤ 45; age ≥ 55; age ≥ 65) were conducted. The positive association between Mg intake and GM and WM volumes was weaker with advancing age. Compared to individuals ≤ 45 years, every additional 1 mg in Mg intake above 350 mg/day was associated with − 0.0001% lower GM volume and − 0.0002% lower WM volume in individuals at age 55 years.

### Sensitivity analysis

Sensitivity analysis was performed to control for Ca and energy intake in assessing the relationship between dietary Mg intake and brain volumes. The significant association between dietary Mg intake and brain volumes remained unchanged after accounting for Ca, indicating that this relationship is independent of Ca levels (Table S12). Furthermore, the association between dietary Mg intake and brain volumes persisted after controlling for energy intake, suggesting that energy intake does not impact this association (Table S12).

Additional analyses stratified by sex were conducted to better characterise sex-specific associations between Mg intake and brain regions (Table S14). Higher dietary Mg intake was significantly associated with larger GM volume, with a stronger association observed in men compared to women.

### Mediation effects of inflammation

**Fig. 1:**
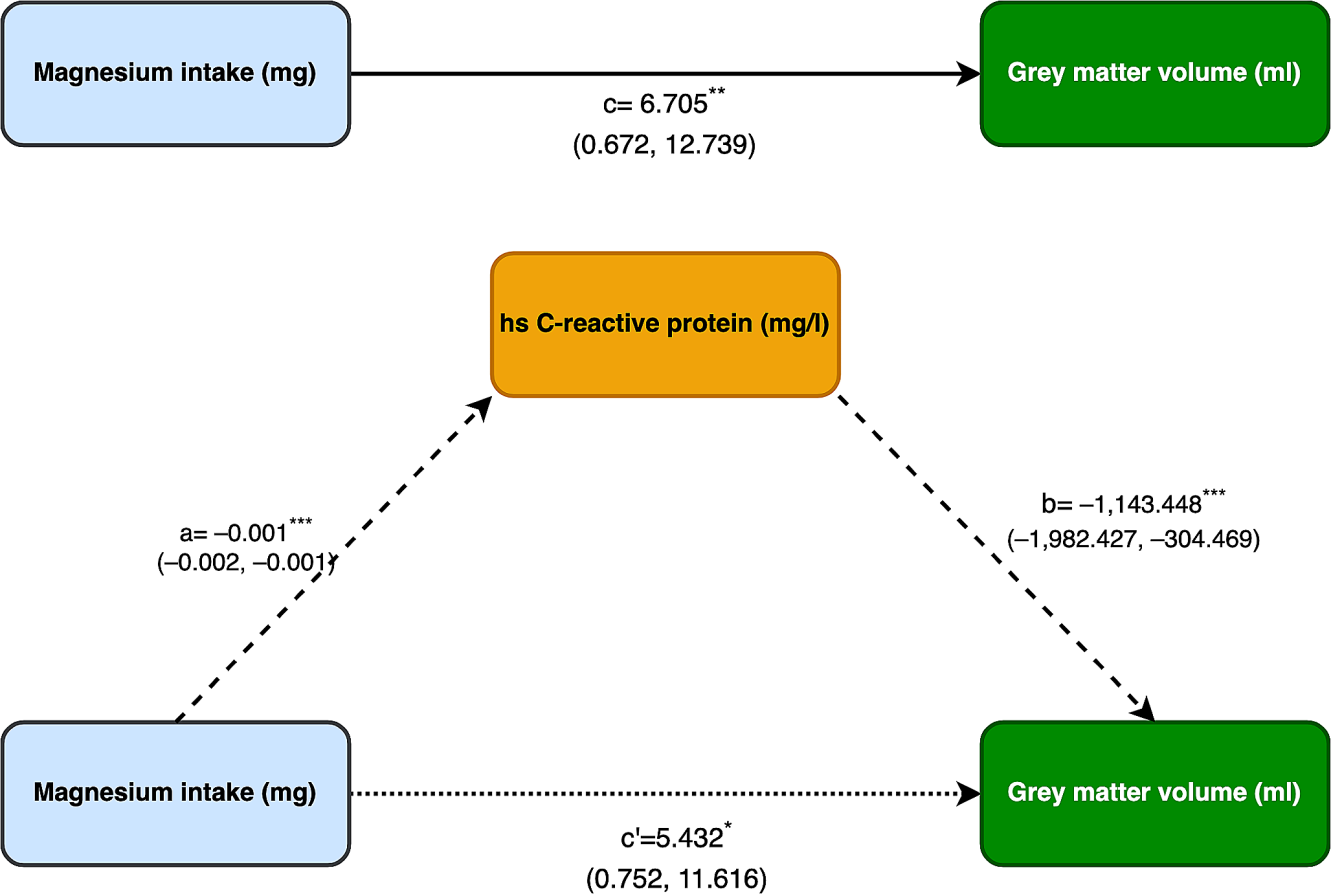
Mediation analysis testing whether the dietary magnesium (Mg) intake on grey matter volume (GM) is mediated through high-sensitivity c-reactive protein (hs-CRP). a = the effect of dietary Mg intake on hs-CRP, b = the effect of hs-CRP on the GM, c = the effect of dietary Mg intake on GM, c’= is the direct effect of dietary Mg intake on GM. The indirect effect was calculated with bootstrapping method (stimulation number = 1000)

The possible mediating role of inflammatory markers on the relationship between dietary Mg intake and brain volumes and WMLs was investigated. Significant mediation was only detected for GM for which the effect of dietary Mg intake was partially-mediated by hs-CRP levels (direct effect, B = 5.432, indirect effect; B = 1.1434) (Fig. 1).

## Discussion

This study produced several important findings. Firstly, dietary Mg intake was found to be associated with reduced inflammation. Secondly, the neuroprotective effect of dietary Mg intake was confirmed. Finally, Mg’s neuroprotective effect was found to be in part mediated by inflammation.

A key finding was that low dietary Mg intake is related to higher levels of inflammation. The findings demonstrate that individuals who consume ~ 15% (50 mg) below the daily recommended intake of Mg (350 mg) have on average a 2.48% higher hs-CRP level, a 0.075% higher leukocyte count, and a 2.59% higher GlycA level. The increase in hs-CRP levels observed in individuals with lower Mg intake is likely due to the elevated expression and secretion of interleukins. This is consistent with findings from a meta-analysis of randomized control trials that found a significant inverse relationship between Mg supplementation and lower CRP and IL-6 levels [25]. Additionally, this interpretation is also supported by our findings that low Mg intake was associated with higher leukocyte counts. Indeed, leucocytes proliferation is up-regulated in the early stages of the immune response and in turn leads to an increased production of interleukins and particularly IL-1 and IL-6, which are known to be implicated in neurodegenerative processes [32, 55]. Moreover, similar inverse association between serum Mg levels and leukocyte count was demonstrated in COVID-19 patients [56]. Therefore, these results are consistent with a plausible mechanistic cascade linking Mg intake to a pro-inflammatory immune response reflected by higher hs-CRP levels.

Furthermore, our study is to our knowledge the first to show that dietary Mg intake is associated with lower GlycA levels. This is important because GlycA is a recently discovered biomarker that has been shown to be associated with atherosclerosis [57–59], cardiovascular events [35–38, 60], and heart failure [26]. Higher GlycA levels have been linked to higher levels of hs-CRP and pro-inflammatory cytokines including IL-6, as well as TNF-α, and fibrinogen [55]. The present findings suggest that the relationship between Mg intake and BP levels may be mediated by the inflammatory biomarker GlycA. This may also partially explain the neuroprotective effect of GlycA in vascular diseases. Further research is needed to investigate the specific mechanisms by which Mg intake affects BP levels possibly via GlycA mediation. Together, the present findings and evidence from the broader literature present a consistent picture of Mg being associated with a lower inflammatory response at different stages of the immunological process.

**Fig. 2:**
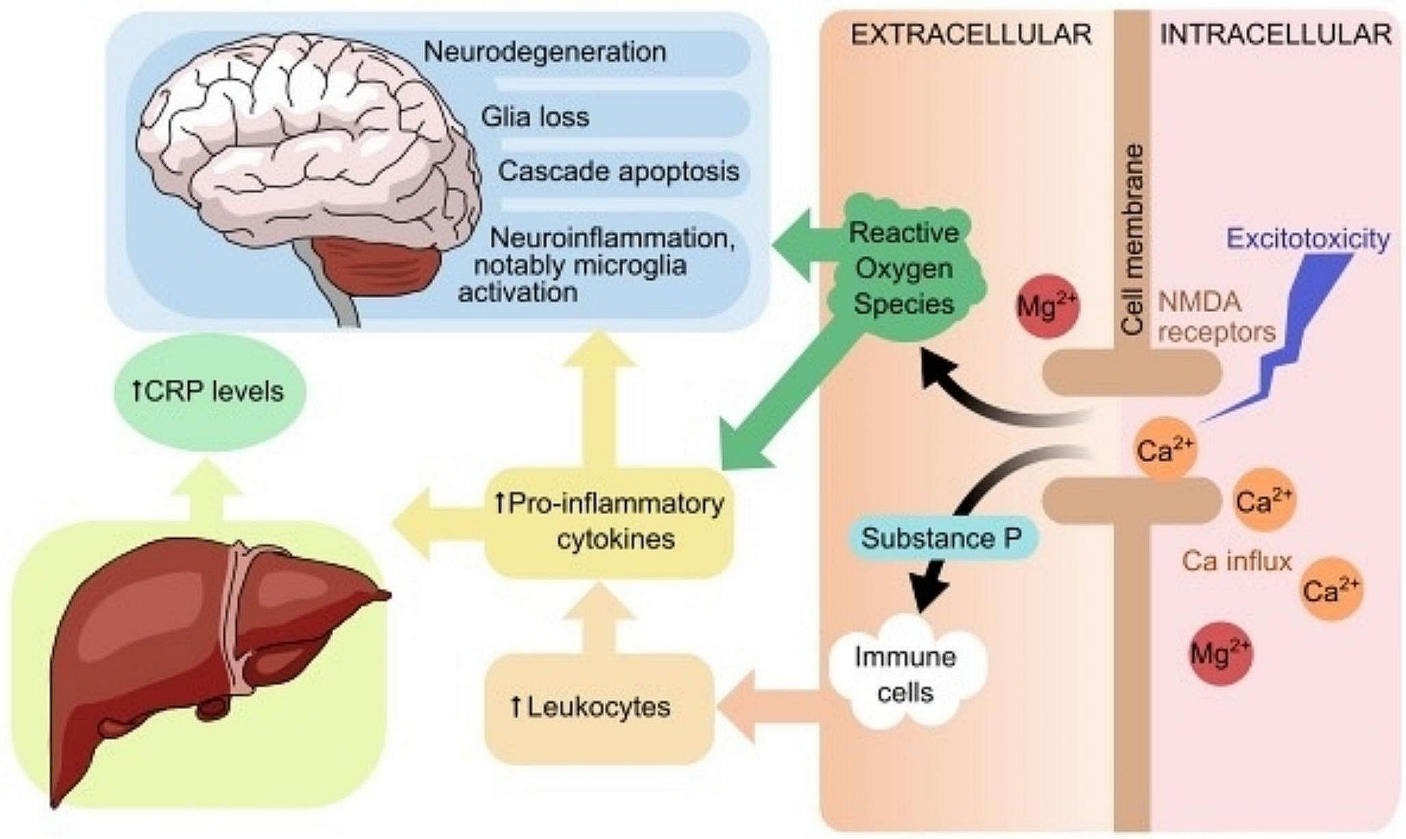
The figure illustrates the cellular mechanisms that connect higher levels of magnesium to a reduction in inflammatory response and improved cerebral health

The novel and the most significant finding of this study is that the neuroprotective effect associated with dietary Mg intake is mediated by lower inflammation. Specifically, lower hs-CRP levels significantly mediated the positive association between higher dietary Mg intake and larger GM volume. Our findings are in line with evidence from animal studies, which have demonstrated a relationship between lower Mg intake and higher neuroinflammation. Research has suggested that a reduction in Mg intake can trigger microglia activation, which may result in an increase in proinflammatory cytokines including IL-6, TNF-α, and nitric oxide [23]. Treatment with Mg has been shown to decrease microglia activation and inhibit TNF-α production in rats [24]. Although the exact mechanisms linking dietary Mg, inflammation, and cerebral health are not yet fully understood, some evidence suggests that a decrease in extracellular Mg ion concentrations could activate macrophages and increase the influx of calcium ions into various types of cells, such as adipocytes, neurons, and peritoneal cells. This, in turn, could lead to hyperexcitability of cells due to overstimulation of N-methyl-D-aspartate (NMDA) receptors [61]. Additionally, the reduction in Mg ions may increase the release of neuromediators such as substance P and neuroinflammatory tachykinins, as well as stimulate pro-inflammatory cytokines such as IL-6, TNF-α, and nitric oxide. The latter can act as signalling molecules and increase the release of CRP from the liver as part of the acute phase response, and may prolong the inflammatory response [62]. On the other hand, higher TNF-α levels further upregulate the production of pro-inflammatory cytokines (e.g. IL-1, IL-6), which has been linked to activation of the apoptosis cascade, glial cell loss, and GM atrophy both in rats experiments and human observational studies [63–65] (Fig. 2). These findings suggest that maintaining a sufficient, and possibly somewhat raised, dietary Mg intake may contribute to decreasing neurodegeneration and therefore protect cerebral health through its anti-inflammatory action. While the effects observed in this study were small, the fact that mediation was detected in an epidemiological context with many uncontrolled factors suggests that the findings are noteworthy and may have been under-estimated. However, it is important to state that further research is needed to confirm these results and to better understand the underlying mechanisms.

Importantly, sensitivity analyses revealed that the association between Mg and GM volume are stronger in men. This is consistent with findings from previous studies which have shown sex related differences in the association between Mg intake and brain volumes [52]. This sex difference may be attributed to hormonal effects, including variations in estrogen and testosterone levels, aligning with Mg’s known role in modulating hormonal pathways and regulating levels in the body [66–68]. Another possible cause may relate to the fact that exposure to lifestyle factors, which is known to differ between men and women, such as physical activity which can differentially impact cardiovascular health [69], and contribute to the observed sex-specific effects. However, it should also be acknowledged that lacking sufficient statistical power for stratified analyses may increase the risk of identifying significant associations within a specific group, and thus may lead to contradictory results between sex interactions in combined models and stratified sex effects. This may lead to confusing and potentially misleading conclusions [9–11]. Therefore, future studies should aim to clarify sex differences in the association between Mg and neurodegeneration.

Nonetheless, it should be noted we did not find any evidence of inflammation playing a mediating role in the association between dietary Mg intake and other brain regions. This may be due to the decreased capacity to detect diffuse effects in smaller brain areas, or attributable to other mechanisms. For example, Mg has been shown to help prevent synaptic loss by blocking the cytotoxic effects of NMDA and therefore, increase neurogenesis and decrease neurodegeneration [70, 71].

Finally, it is noteworthy that the anti-inflammatory benefits of dietary Mg intake appear to be weakened in the presence of cardiovascular risk factors such as smoking, high cholesterol levels, and hypertension. Indeed, in those who presented with these risk factors the association between higher Mg and lower inflammation was weaker than in those who did not. In contrast, these associations were stronger in individuals taking BP medication. The reasons for these interactions are unclear and need further investigation. It is possible that smoking in particular may interfere with the absorption and utilization of Mg in the body [72], leading to a weaker anti-inflammatory response. It is also possible that the strong pro-inflammatory and/or neurodegenerative effects of cardiovascular risk factors may have obscured or dampened an Mg effect. In contrast, BP medication may work synergistically with dietary Mg intake in reducing inflammation [73]. It is important to highlight that the exact mechanisms behind these observations are not clear and further research is needed to identify them.

### Limitation and strength

This study has a number of limitations but also significant strengths. Mg intake was assessed indirectly, using food frequency questionnaires [41, 42]. This method is known to be reliable [74], although it is also linked to more measurement error, which may have reduced our ability to detect some relationships. Nonetheless, nutrition was assessed multiple times during the follow-up period, which is likely to have reduced any recall bias [74]. Another possible conceptual limitation is the use of a single nutrient investigation, which may oversimplify the complex interplay of nutrients within the human diet [75–77]. The health benefits associated with magnesium-rich foods, such as, green vegetables, nuts, seeds, and unrefined grains, include anti-inflammatory, antioxidant, as well as other effects that are only partly attributable to its Mg content [78] and which therefore cannot be estimated in single nutrient analyses. On the other hand, this approach provides more specific estimates of dose-effects which can contribute to systematic review and may be useful in informing clinical practice and population health interventions [9]. Another limitation is the lack of availability of more detailed inflammatory measures, in particular IL-1 and IL-6 which have been consistently implicated in neurodegenerative processes [27, 79], as well as the biological pathways linking Mg to inflammatory processes [80]. lastly, due to the observational nature of our study it is difficult to establish a conclusive cause-and-effect relationship.

A particular strength is that this study examined a very large number of participants with enough statistical power to test hypothesised associations and mediation while adjusting for a large number of cardiovascular factors. To our knowledge, it was also the first to use neuroimaging and structural brain measures in humans to examine the mediation effect of dietary Mg intake on cerebral health through the inflammatory process.

## Conclusion

In summary, the current findings provide convergent evidence suggesting that the anti-inflammatory effects of dietary Mg in middle-to early old-age adults may have a neuroprotective effect. Thus, these findings are a promising step towards understanding the potential benefits of dietary Mg intake, and in gauging the value Mg supplementation and dietary advice may contribute to interventions aimed at reducing inflammation and promoting cerebral health in the general population.

### Electronic supplementary material

Below is the link to the electronic supplementary material.

Supplementary Material 1

## Data Availability

UK Biobank is an open access resource accessible to confirmed researchers upon request (ukbiobank.ac.uk/).
