# Supplementary material for: Association between dietary magnesium intake, inflammation, and neurodegeneration
Source: Eur J Nutr. 2024 Apr 10;63(5):1807–18. doi: 10.1007/s00394-024-03383-1 (PMC11329609; doi:10.1007/s00394-024-03383-1)
Supplement: Supplementary file 1 — Supplementary Material 1 [file 394_2024_3383_MOESM1_ESM.pdf]

**Title Page**: Association between dietary magnesium intake, inflammation, and neurodegeneration.

**Running Title:** Magnesium, inflammation, and brain volumes

**Authors:**

Ms. Khawlah Alateeq, MMagReson[1,2*]

Dr. Erin I. Walsh, PhD[1]

Dr. Ananthan Ambikairajah PhD[3]

Prof. Nicolas Cherbuin, PhD[1]

**Affiliations:**

National Centre for Epidemiology and Population Health, Australian National University, Canberra, Australia.[1]

Radiological Science, College of Applied Medical Science, King Saud University, Riyadh, Saudi Arabia.[2]

Discipline of Psychology, Faculty of Health, University of Canberra, Canberra, ACT 2617, Australia[3]

***Correspondence:**

Khawlah Alateeq, National Centre for Epidemiology and Population Health, 54 Mills Road, Australian National University, Canberra, ACT 2601, Australia.

Tel: (612) 61252120; Email: Khawlah.alateeq@anu.edu.au

ORCID: 0000-0002-0792-7357

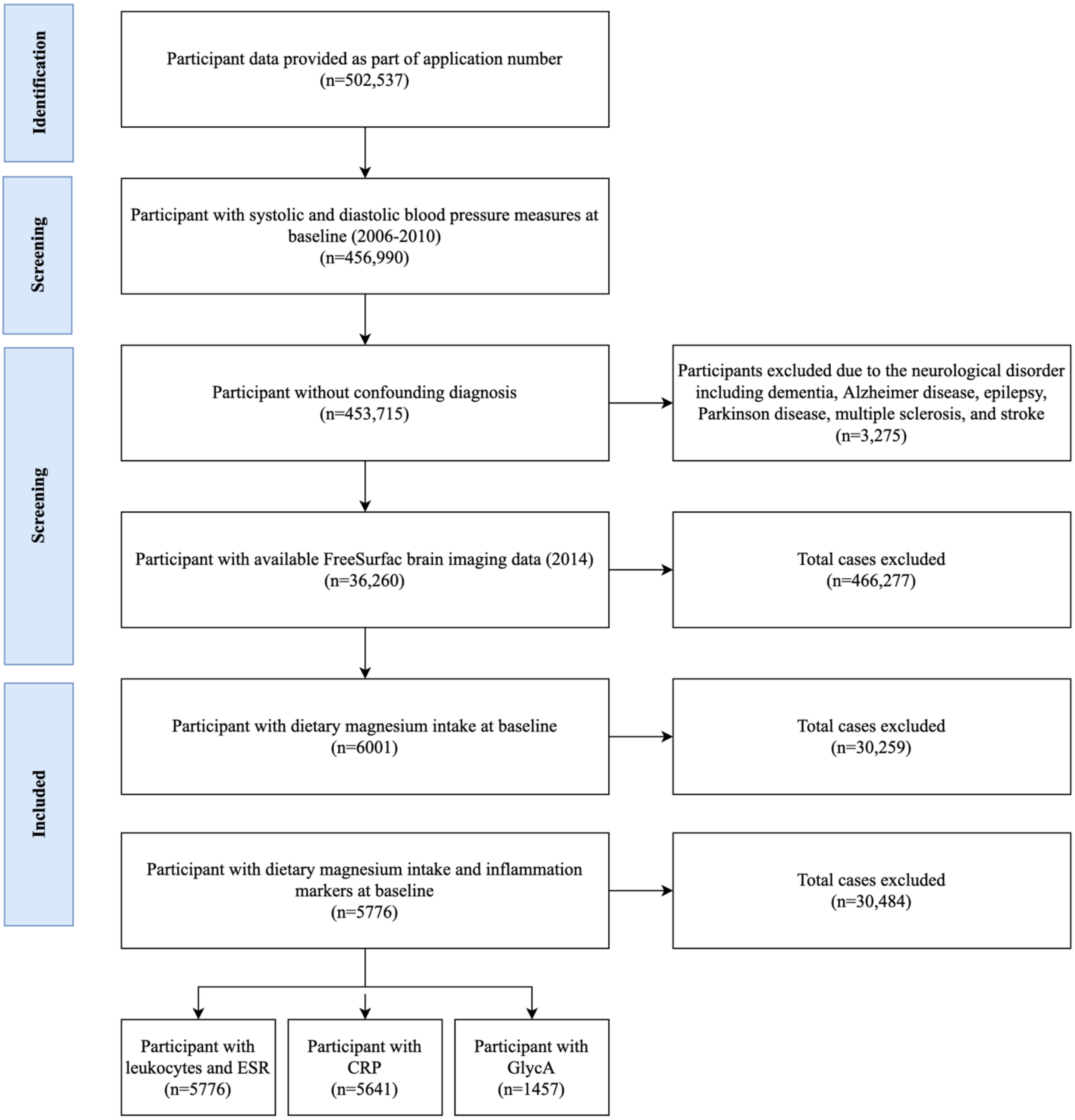


FigureS1. Flow chart of the participants selection process.

**Table S.** Demographic and health characteristics of included and excluded participants.

**Selected vs non-selected**

| Measures | Whole Sample | Not selected | Selected | T/chi-sq Test (P value) |
|---|---|---|---|---|
| Age, year (SD) | 56.53 (8.10) | 56.43 (8.09) | 56.54 (8.10) | -2.81 (0.005) |
| SBP, mmHg (SD) | 139.74 (19.70) | 139.97 (20.35) | 139.73 (19.68) | 1.20 (0.230) |
| DBP, mmHg (SD) | 82.21 (10.70) | 82.66 (11.19) | 82.19 (10.69) | 4.36 (0.000) |
| BMI, kg/m2 (SD) | 27.43 (4.80) | 27.77 (5.21) | 27.40 (4.76) | 14.40 (0.000) |
| Cholesterol, mmol/L | 5.69 (1.14) | 5.70 (1.16) | 5.69 (1.14) | 1.14 (0.255) |
| HDL mmol/L | 1.45 (0.38) | 1.43 (0.38) | 1.45 (0.38) | -7.35 (0.000) |
| Sex, n (Male%) | 229134 (50.00%) | 20295 (44.56%) | 208839 (45.70%) | 21.63 (0.000) |
| Smoking, n (%), | 301690 (59.69%) | 26604 (59.16%) | 272187 (59.86%) | 8.41 (0.004) |

Significance: $p<0.05$

There were 10 (0.02%) participants missing data for diabetes, 17 (0.04%) participants missing data for alcohol conception, 43 (0.11%) participants missing data for body mass index , 77 (0.2%) participants missing data for smoking status, 335 (0.9%), participants missing data for antihypertensive medication, 2096 (5.7%) participants missing data for total cholesterol, 2540 (7%) participants missing data for education, 5142 (14.1%) participants missing data for high-density lipoprotein cholesterol, and 5177 (14.2%) participants missing data for physical activity. All missing values were imputed.

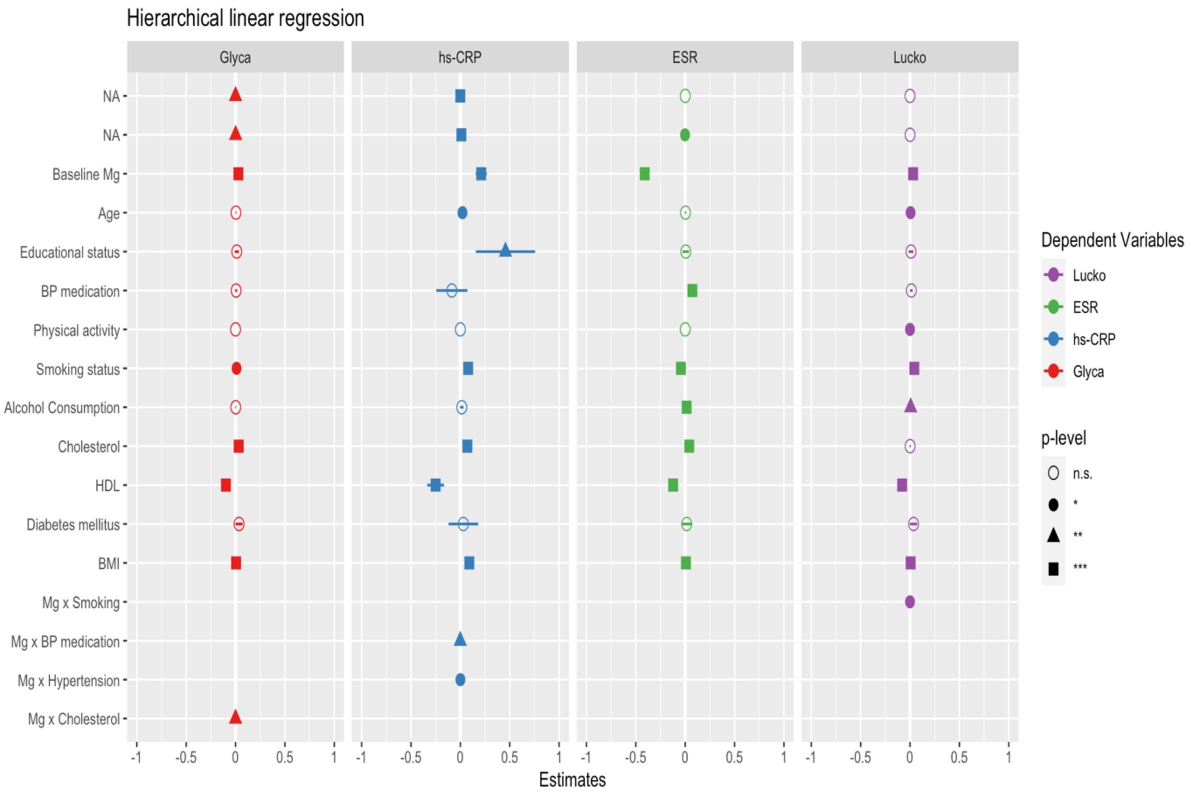

Figure S2 shows the results of hierarchical regression analysis exploring the association between dietary magnesium (Mg) intake and inflammatory markers (leukocytes, erythrocyte, C-reactive protein (hs-CRP), and Glycoprotein acetylation (GlycA)) in the UK Biobank study. The data is presented as unstandardized Beta correlation with the standard error (+/- SE), where Beta represents the effect size of a 1 mg increase in Mg intake, expressed in SD units of the dependent variable. Model 1 was adjusted for main covariates, including age, sex, and education. Model 2 was further adjusted for BP medication, HDL cholesterol, diabetes mellitus, smoking status, higher education, physical activity, alcohol intake and body mass index (BMI). Model 3 tested the two-way interactions between Mg and smoking, Mg and BP medication, Mg and hypertension, and Mg and cholesterol level. Note that error bars represent standard errors from the same model. Significance levels were indicated as *$p < 0.05$, **$p < 0.01$, and ***$p < 0.001$.

**Table S1. Association between dietary Mg intake and inflammation markers.**

| | Leukocytes ($10^9$ cells/L) | Erythrocyte ($10^{12}$ cells/L) | hs-CRP Level (mg/L) | GlycA (mmol/L) |
|---|---|---|---|---|
| Mg | -0.0001$^{*}$ (0.00003) | -0.00001 (0.00003) | -0.001$^{****}$ (0.0001) | -0.0004$^{***}$ (0.0001) |
| Age | 0.001 (0.0004) | -0.001$^{**}$ (0.001) | 0.010$^{****}$ (0.002) | 0.001$^{***}$ (0.0004) |
| Women | 0.031$^{****}$ (0.007) | -0.410$^{****}$ (0.010) | 0.211$^{****}$ (0.029) | 0.029$^{****}$ (0.006) |
| Educational status | 0.006$^{**}$ (0.003) | 0.003 (0.004) | 0.021$^{**}$ (0.011) | 0.004$^{*}$ (0.002) |
| BP medication | 0.010 (0.012) | 0.006 (0.017) | 0.457$^{***}$ (0.153) | 0.012 (0.011) |
| Hypertension | 0.013$^{*}$ (0.007) | 0.073$^{****}$ (0.010) | -0.087 (0.080) | 0.006 (0.006) |
| Physical activity | -0.00000$^{**}$ (0.00000) | -0.00000 (0.00000) | -0.00001 (0.00001) | -0.00000 (0.00000) |
| Smoking status | 0.044$^{****}$ (0.005) | -0.043$^{****}$ (0.007) | 0.079$^{****}$ (0.021) | 0.010$^{**}$ (0.004) |
| Alcohol Consumption | 0.007$^{***}$ (0.002) | 0.015$^{****}$ (0.003) | 0.014 (0.009) | 0.002 (0.002) |
| Cholesterol | 0.001 (0.003) | 0.041$^{****}$ (0.004) | 0.070$^{****}$ (0.012) | 0.032$^{****}$ (0.003) |
| HDL | -0.080$^{****}$ (0.011) | -0.122$^{****}$ (0.015) | -0.251$^{****}$ (0.043) | -0.098$^{****}$ (0.009) |
| Diabetes mellitus | 0.036$^{*}$ (0.019) | 0.016 (0.026) | 0.030 (0.077) | 0.036$^{*}$ (0.018) |
| BMI | 0.007$^{****}$ (0.001) | 0.008$^{****}$ (0.001) | 0.090$^{****}$ (0.003) | 0.005$^{****}$ (0.001) |
| Mg x Smoking status | 0.0001$^{**}$ (0.00004) | | | |
| Mg x BP medication | | | -0.001$^{***}$ (0.0004) | |
| Mg x Hypertension | | | 0.0004$^{**}$ (0.0002) | |
| Mg x Cholesterol | | | | 0.0001$^{***}$ (0.00002) |
| Constant | -4.292$^{****}$ (0.038) | 4.536$^{****}$ (0.053) | -2.826$^{****}$ (0.160) | 0.503$^{****}$ (0.032) |
| Observations | 5,766 | 5,766 | 5,468 | 1,417 |

The statistical significance was indicated by *p<0.05, **p<0.01, and ***p<0.001. SE stands for standard error, Mg refers to magnesium, and GlycA is a measure of glycoprotein acetylation HDL, high-density lipoprotein; BMI, body mass index. The data represents unstandardized Beta correlation with the standard error (+/- SE), where Beta represents the effect size of a 1 mg increase in Mg intake, expressed in SD units of the dependent variable. Note that the hierarchical analysis results of the association between dietary Mg intake and inflammatory markers, including leukocytes, erythrocyte count, and GlycA were obtained from three models. Model 1 was adjusted for main covariates, including age, sex, and education. Model 2 was further adjusted for other covariates, such as antihypertensive medication, HDL, cholesterol, diabetes mellitus, smoking status, higher education, physical activity, alcohol intake and BMI. Model 3 also examined the two-way interactions between Mg and cholesterol level.

.

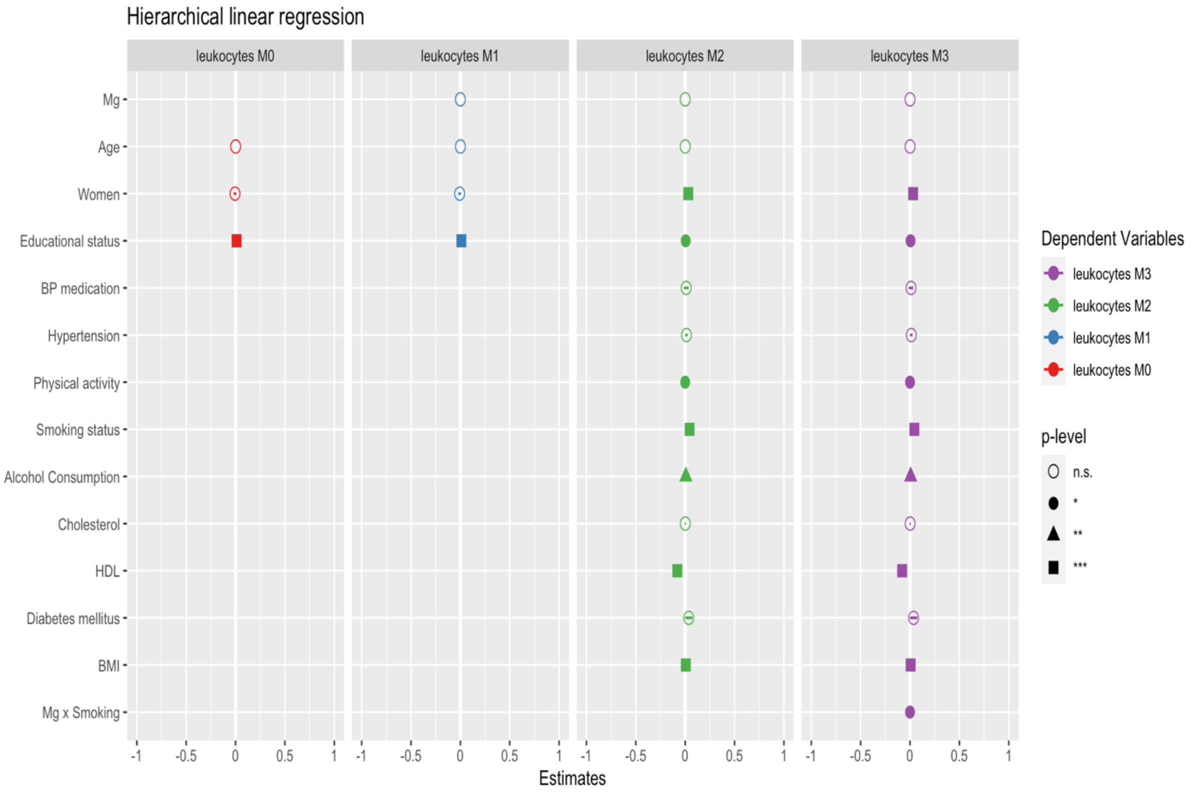


Figure S3. The figure presents the results of hierarchical regression analysis examining the association between dietary magnesium intake and leukocyte count in the UK Biobank study. The data is presented as unstandardized Beta correlation with the standard error (+/- SE), where Beta represents the effect size of a 1 mg increase in Mg intake, expressed in SD units of the dependent variable. Model 1 was adjusted for main covariates, including age, sex, and education. Model 2 was further adjusted for antihypertensive medication, HDL cholesterol, diabetes mellitus, smoking status, higher education, physical activity, alcohol intake and body mass index (BMI). Model 3 tested the two-way interaction between magnesium intake and smoking status. Note: Error bars represent standard errors from the same model. Significance levels are indicated as * $p < 0.05$; ** $p < 0.01$; *** $p < 0.001$.

Table S2. The Association Between Dietary Magnesium Intake and Leukocytes

| | Leukocytes | | | |
|---|---|---|---|---|
| | Model 0 | Model 1 | Model 2 | Model 3 |
| Mg | | -0.00004 (0.00003) | -0.00002 (0.00003) | -0.0001* (0.00003) |
| Age | 0.001 (0.0004) | 0.001 (0.0004) | 0.001 (0.0004) | 0.001 (0.0004) |
| Women | -0.006 (0.006) | -0.007 (0.006) | 0.031**** (0.007) | 0.031**** (0.007) |
| Educational status | 0.010**** (0.003) | 0.010**** (0.003) | 0.005** (0.003) | 0.006** (0.003) |
| BP medication | | | 0.010 (0.012) | 0.010 (0.012) |
| Hypertension | | | 0.013* (0.007) | 0.013* (0.007) |
| Physical activity | | | -0.00000** (0.00000) | -0.00000** (0.00000) |
| Smoking status | | | 0.045**** (0.005) | 0.044**** (0.005) |
| Alcohol Consumption | | | 0.007*** (0.002) | 0.007*** (0.002) |
| Cholesterol | | | 0.001 (0.003) | 0.001 (0.003) |
| HDL | | | -0.080**** (0.011) | -0.080**** (0.011) |
| Diabetes mellitus | | | 0.036* (0.019) | 0.036* (0.019) |
| BMI | | | 0.007**** (0.001) | 0.007**** (0.001) |
| Mg x Smoking | | | | 0.0001** (0.00004) |
| Constant | -4.184**** (0.024) | -4.184**** (0.024) | -4.293**** (0.038) | -4.292**** (0.038) |
| Observations | 5,776 | 5,776 | 5,766 | 5,766 |

Statistical significance was indicated by *p < 0.05, **p < 0.01, and ***p < 0.001. Abbreviations: SE, standard error; Mg, magnesium; HDL, high-density lipoprotein; BMI, body mass index. The data represents unstandardized Beta correlation with the standard error (+/- SE), where Beta represents the effect size of a 1 mg increase in Mg intake, expressed in SD units of the dependent variable. Note that the hierarchical analysis results of the association between dietary Mg intake and leukocytes were obtained from three models. Model 1 was adjusted for main covariates, including age, sex, and education. Model 2 was further adjusted for additional covariates, including antihypertensive medication, HDL cholesterol, diabetes mellitus, smoking status, higher education, physical activity, alcohol intake, and BMI. Model 3 tested the two-way interaction between Mg and smoking status.

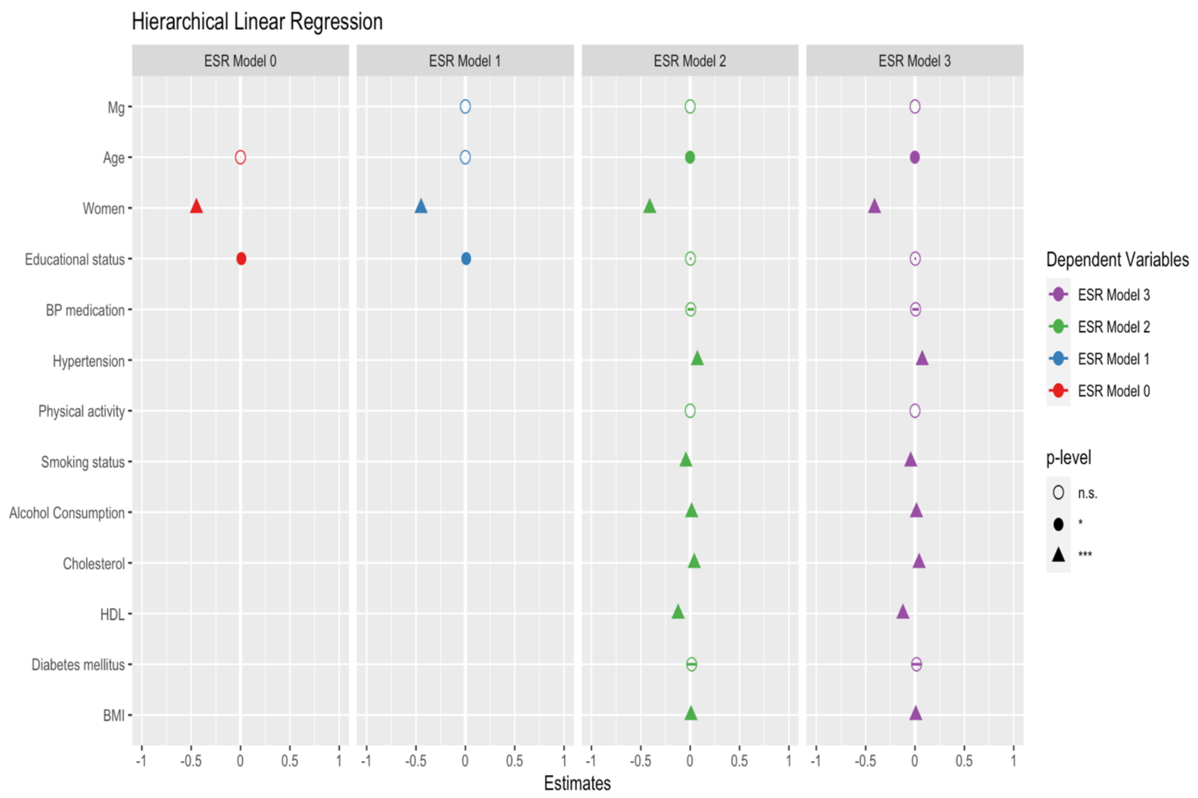


Figure S4. The figure presents the results of hierarchical regression analysis examining the association between dietary magnesium intake and erythrocyte count in the UK Biobank study The data is presented as unstandardized Beta correlation with the standard error (+/- SE), where Beta represents the effect size of a 1 mg increase in Mg intake, expressed in SD units of the dependent variable. Model 1 was adjusted for the main covariates including age, sex, and education. Model 2 was additionally adjusted for antihypertensive medication, HDL, cholesterol, diabetes mellitus, smoking status, higher education, physical activity, alcohol intake and body mass index (BMI). Model 3 was additionally tested for the two-way interactions between magnesium intake and the covariates. Error bars represent standard error from the same model. Significance levels were indicated as *$p < 0.05$, **$p < 0.01$, and ***$p < 0.001$

Table S3. The association between dietary magnesium intake and erythrocytes in the UK Biobank Study.

| | Erythrocyte | | | |
|---|---|---|---|---|
| | Model 0 | Model 1 | Model 2 | Model 3 |
| Mg | | -0.00004 (0.00004) | -0.00001 (0.00003) | -0.00001 (0.00003) |
| Age | -0.001 (0.001) | -0.001 (0.001) | -0.001$^{**}$ (0.001) | -0.001$^{**}$ (0.001) |
| Women | -0.447$^{****}$ (0.009) | -0.449$^{****}$ (0.009) | -0.410$^{****}$ (0.010) | -0.410$^{****}$ (0.010) |
| Educational status | 0.008$^{**}$ (0.004) | 0.008$^{**}$ (0.004) | 0.003 (0.004) | 0.003 (0.004) |
| BP medication | | | 0.006 (0.017) | 0.006 (0.017) |
| Hypertension | | | 0.073$^{****}$ (0.010) | 0.073$^{****}$ (0.010) |
| Physical activity | | | -0.00000 (0.00000) | -0.00000 (0.00000) |
| Smoking status | | | -0.043$^{****}$ (0.007) | -0.043$^{****}$ (0.007) |
| Alcohol Consumption | | | 0.015$^{****}$ (0.003) | 0.015$^{****}$ (0.003) |
| Cholesterol | | | 0.041$^{****}$ (0.004) | 0.041$^{****}$ (0.004) |
| HDL | | | -0.122$^{****}$ (0.015) | -0.122$^{****}$ (0.015) |
| Diabetes mellitus | | | 0.016 (0.026) | 0.016 (0.026) |
| BMI | | | 0.008$^{****}$ (0.001) | 0.008$^{****}$ (0.001) |
| Constant | 4.808$^{****}$ (0.034) | 4.808$^{****}$ (0.034) | 4.536$^{****}$ (0.053) | 4.536$^{****}$ (0.053) |
| Observations | 5,776 | 5,776 | 5,766 | 5,766 |

The statistical significance levels were indicated as *p < 0.05, **p < 0.01, and ***p < 0.001. Abbreviations: SE, standard error; Mg, magnesium; HDL, high-density lipoprotein; BMI, body mass index. The data presented here shows unstandardized Beta correlation with the standard error (+/- SE), where Beta represents the effect size of a 1 mg increase in Mg intake, expressed in SD units of the dependent variable. It should be noted that the hierarchical analysis results of the association between dietary Mg intake and erythrocytes were obtained from three models. Model 1 was adjusted for the main covariates including age, sex, and education. Model 2 was additionally adjusted for other covariates, such as antihypertensive medication, HDL cholesterol, diabetes mellitus, smoking status, higher education, physical activity, and alcohol intake. Finally, Model 3 tested the two-way interactions between Mg x covariates

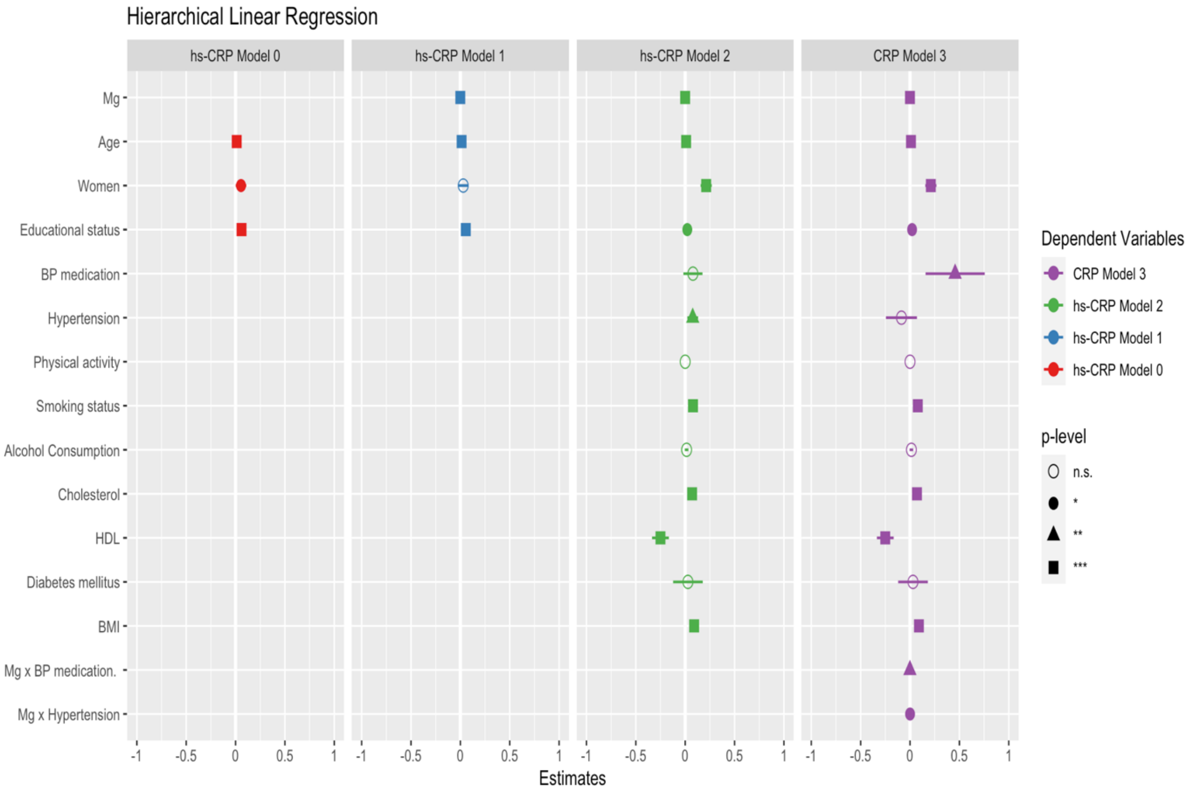


Figure S5 The figure presents the results of hierarchical regression analysis examining the association between dietary magnesium (Mg) intake and high-sensitivity C-reactive protein (hs-CRP) levels in the UK Biobank study. The data is presented as unstandardized Beta correlation with the standard error (+/- SE), where Beta represents the effect size of a 1 mg increase in Mg intake, expressed in SD units of the dependent variable. Model 1 was adjusted for key covariates including age, sex, and education. Model 2 additionally controlled for antihypertensive medication, HDL cholesterol, diabetes mellitus, smoking status, higher education, physical activity, and body mass index (BMI). Model 3 tested two-way interactions between Mg x Hypertension and Mg x BP medication. Error bars represent the standard error from the same model. Statistical significance was indicated by *p < 0.05, **p < 0.01, and ***p < 0.001

Table S4. The association between dietary magnesium intake and high-sensitivity c-reactive protein (hs-CRP) levels in the UK Biobank Study

| | Hs-CRP | | | |
|---|---|---|---|---|
| | Model 0 | Model 1 | Model 2 | Model 3 |
| Mg | | -0.001$^{****}$ (0.0001) | -0.001$^{****}$ (0.0001) | -0.001$^{****}$ (0.0001) |
| Age | 0.012$^{****}$ (0.002) | 0.012$^{****}$ (0.002) | 0.010$^{****}$ (0.002) | 0.010$^{****}$ (0.002) |
| Women | 0.055$^{**}$ (0.027) | 0.029 (0.027) | 0.213$^{****}$ (0.029) | 0.211$^{****}$ (0.029) |
| Educational status | 0.061$^{****}$ (0.012) | 0.055$^{****}$ (0.012) | 0.021$^{**}$ (0.011) | 0.021$^{**}$ (0.011) |
| BP medication | | | 0.078 (0.049) | 0.457$^{***}$ (0.153) |
| Hypertension | | | 0.076$^{***}$ (0.028) | -0.087 (0.080) |
| Physical activity | | | -0.00001 (0.00001) | -0.00001 (0.00001) |
| Smoking status | | | 0.079$^{****}$ (0.021) | 0.079$^{****}$ (0.021) |
| Alcohol Consumption | | | 0.014 (0.009) | 0.014 (0.009) |
| Cholesterol | | | 0.070$^{****}$ (0.012) | 0.070$^{****}$ (0.012) |
| HDL | | | -0.251$^{****}$ (0.043) | -0.251$^{****}$ (0.043) |
| Diabetes mellitus | | | 0.028 (0.077) | 0.030 (0.077) |
| BMI | | | 0.091$^{****}$ (0.003) | 0.090$^{****}$ (0.003) |
| Mg x BP medication | | | | -0.001$^{***}$ (0.0004) |
| Mg x Hypertension | | | | 0.0004$^{**}$ (0.0002) |
| Constant | -0.611$^{****}$ (0.104) | -0.372$^{****}$ (0.111) | -2.871$^{****}$ (0.157) | -2.826$^{****}$ (0.160) |
| Observations | 5,477 | 5,477 | 5,468 | 5,468 |

"The statistical significance levels were indicated as *p < 0.05, **p < 0.01, and ***p < 0.001. Abbreviations: SE, standard error; Mg, magnesium; hs-CRP, high-sensitive C-reactive protein; HDL, high-density lipoprotein; BMI, body mass index. The data is presented as unstandardized Beta correlation with the standard error (+/- SE), where Beta represents the effect size of a 1 mg increase in Mg intake, expressed in SD units of the dependent variable. It should be noted that the hierarchical analysis results of the association between dietary Mg intake and erythrocytes were obtained from three models. Model 1 was adjusted for the main covariates including age, sex, and education. Model 2 was additionally adjusted for other covariates, such as antihypertensive medication, HDL cholesterol, diabetes mellitus, smoking status, higher education, physical activity, and alcohol intake. Finally, Model 3 tested two-way interactions between Mg x Hypertension and Mg x BP medication.

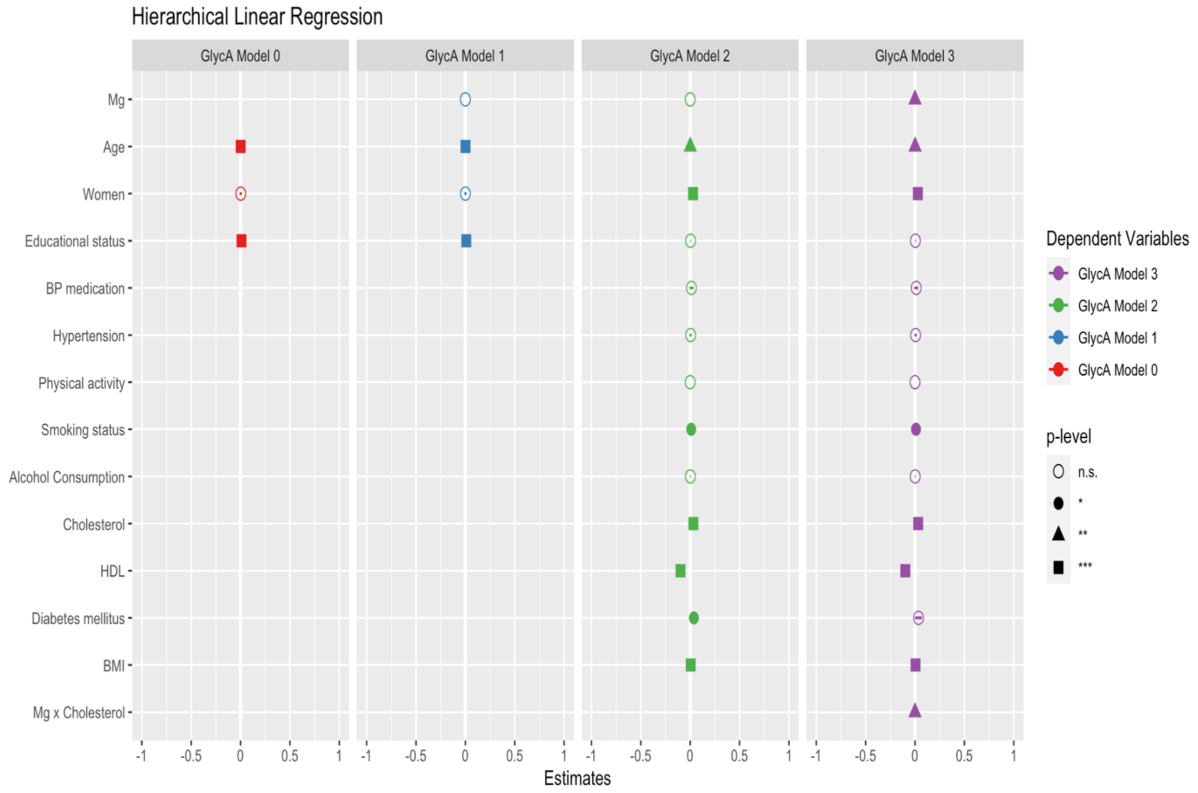


Figure S6 The figure presents the results of hierarchical regression analysis examining the association between dietary magnesium (Mg) intake and glycoprotein acetylation (GlycA) in the UK Biobank study. The data is presented as unstandardized Beta correlation with the standard error (+/- SE), where Beta represents the effect size of a 1 mg increase in Mg intake, expressed in SD units of the dependent variable. Model 1 adjusted for age, sex, and education. Model 2 additionally adjusted for antihypertensive medication, high-density lipoprotein (HDL) cholesterol, diabetes mellitus, smoking status, higher education, physical activity, alcohol intake, and body mass index (BMI). Model 3 tested two-way interactions between Mg x cholesterol level. Statistical significance was denoted by *p<0.05, **p<0.01, and ***p<0.001.

Table S5 the association between dietary magnesium intake and Glycoprotein Acetylation (GlycA) in the UK Biobank Study.

| | GlycA | | | |
|---|---|---|---|---|
| | Model 0 | Model 1 | Model 2 | Model 3 |
| Mg | | -0.00004$^{*}$ (0.00002) | -0.00002 (0.00002) | -0.0004$^{***}$ (0.0001) |
| Age | 0.001$^{****}$ (0.0004) | 0.001$^{****}$ (0.0004) | 0.001$^{***}$ (0.0004) | 0.001$^{***}$ (0.0004) |
| Women | 0.002 (0.006) | 0.001 (0.006) | 0.029$^{****}$ (0.006) | 0.029$^{****}$ (0.006) |
| Educational status | 0.009$^{****}$ (0.003) | 0.009$^{****}$ (0.003) | 0.004$^{*}$ (0.002) | 0.004$^{*}$ (0.002) |
| BP medication | | | 0.013 (0.011) | 0.012 (0.011) |
| Hypertension | | | 0.005 (0.006) | 0.006 (0.006) |
| Physical activity | | | -0.00000 (0.00000) | -0.00000 (0.00000) |
| Smoking status | | | 0.010$^{**}$ (0.005) | 0.010$^{**}$ (0.004) |
| Alcohol Consumption | | | 0.002 (0.002) | 0.002 (0.002) |
| Cholesterol | | | 0.032$^{****}$ (0.003) | 0.032$^{****}$ (0.003) |
| HDL | | | -0.098$^{****}$ (0.009) | -0.098$^{****}$ (0.009) |
| Diabetes mellitus | | | 0.037$^{**}$ (0.018) | 0.036$^{*}$ (0.018) |
| BMI | | | 0.006$^{****}$ (0.001) | 0.005$^{****}$ (0.001) |
| Mg x Cholesterol | | | | 0.0001$^{***}$ (0.00002) |
| Constant | 0.682$^{****}$ (0.022) | 0.681$^{****}$ (0.022) | 0.497$^{****}$ (0.032) | 0.503$^{****}$ (0.032) |
| Observations | 1,419 | 1,419 | 1,417 | 1,417 |

The statistical significance levels were indicated as *p < 0.05, **p < 0.01, and ***p < 0.001. Abbreviations: SE, standard error; Mg, magnesium; GlycA, glycoprotein acetylation; HDL, high-density lipoprotein; BMI, body mass index. The data is presented as unstandardized Beta correlation with the standard error (+/- SE), where Beta represents the effect size of a 1 mg increase in Mg intake, expressed in SD units of the dependent variable. It should be noted that the hierarchical analysis results of the association between dietary Mg intake and erythrocytes were obtained from three models. Model 1 was adjusted for the main covariates including age, sex, and education. Model 2 was additionally adjusted for other covariates, such as antihypertensive medication, HDL cholesterol, diabetes mellitus, smoking status, higher education, physical activity, and alcohol intake. Finally, Model 3 was additionally tested for the two-way interactions between Mg x Cholesterol level.

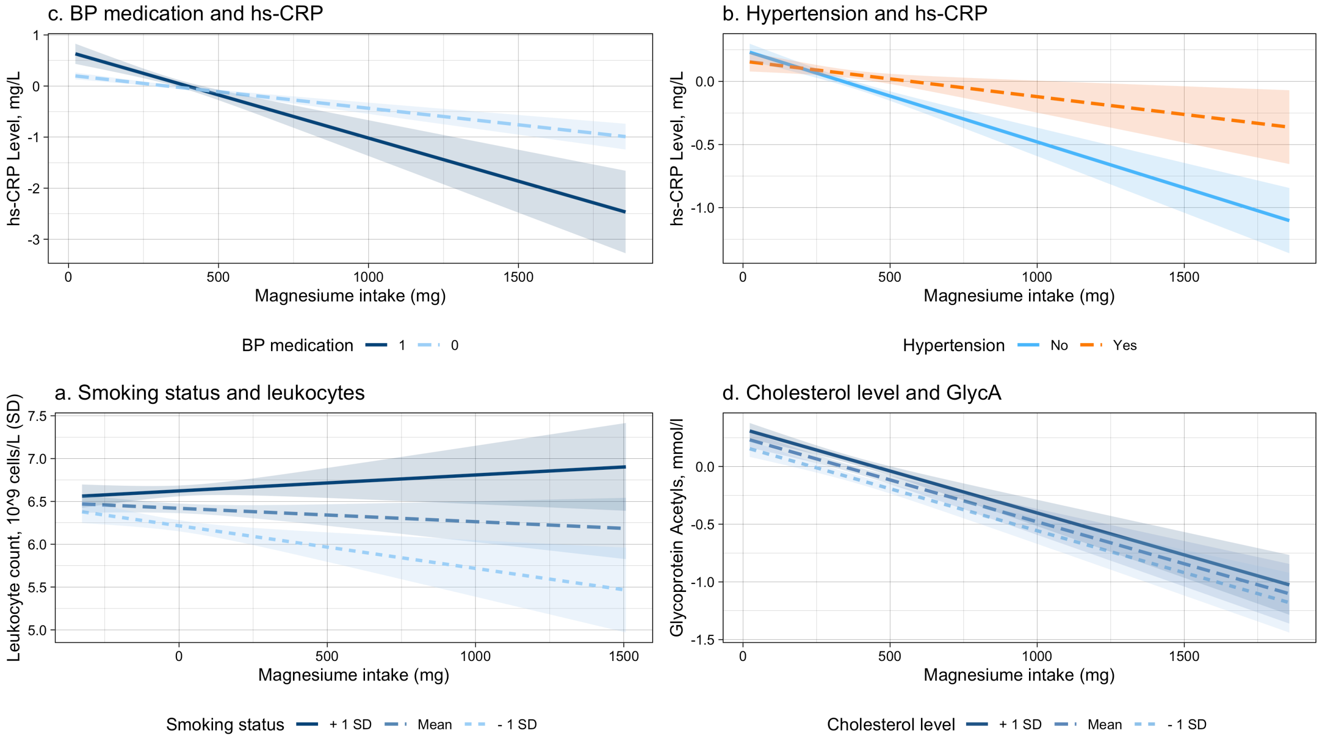


Figure S7. The interaction effects between a) dietary magnesium intake and smoking status in predicting the leukocytes, b) between dietary magnesium intake and Hypertension, BP medication, in predicting the C-reactive protein (hs-CRP) and c) between dietary magnesium intake and cholesterol level in predicting the GlycA. Note. the shadow represents the 95% confidence intervals (CI).

Table S6. The association between dietary Mg intake and brain volumes in the UK Biobank Study.

| | Gray matter volume ($mm^3$) | White matter volume ($mm^3$) | Left hippocampal volume ($mm^3$) | Right hippocampal volume ($mm^3$) | White matter lesions ($mm^3$) |
|---|---|---|---|---|---|
| Mg | 70.006*** (22.615) | 58.106** (23.024) | 0.020 (0.033) | 0.062* (0.033) | 0.00003 (0.0001) |
| Age | -1,567.023**** (53.231) | -1,084.587**** (54.193) | -16.450**** (0.570) | -15.261**** (0.576) | 0.041**** (0.001) |
| Women | -15,418.990**** (1,037.518) | -6,914.226**** (1,056.271) | -35.749*** (11.141) | -25.528** (11.276) | 0.075**** (0.019) |
| Educational status | -416.215 (330.058) | 248.022 (336.024) | -2.913 (3.544) | -0.976 (3.587) | 0.004 (0.006) |
| ICV | 0.304**** (0.003) | 0.311**** (0.003) | 0.001**** (0.00003) | 0.001**** (0.00003) | 0.00000**** (0.00000) |
| BP medication | -4,394.743*** (1,521.193) | -791.655 (1,548.689) | -25.032 (16.335) | -25.334 (16.532) | 0.135**** (0.029) |
| Hypertension | -1,678.662* (857.215) | -553.288 (872.709) | 16.368* (9.205) | 9.619 (9.316) | 0.119**** (0.016) |
| Physical activity | -0.047 (0.167) | 0.313* (0.170) | -0.001 (0.002) | 0.001 (0.002) | 0.00000 (0.00000) |
| Smoking status | -2,484.141**** (641.480) | -572.120 (653.075) | -20.957*** (6.888) | -20.611*** (6.971) | 0.053**** (0.012) |
| Alcohol Consumption | 103.314 (290.337) | 337.812 (295.585) | 1.949 (3.117) | 0.170 (3.155) | 0.001 (0.005) |
| Cholesterol | 2,389.700**** (384.387) | 2,022.601**** (391.335) | 15.351**** (4.127) | 16.573**** (4.177) | -0.019*** (0.007) |
| HDL | -2,391.177* (1,363.636) | -2,245.070 (1,388.284) | -63.564**** (14.643) | -76.043**** (14.820) | 0.016 (0.026) |
| Diabetes mellitus | -9,080.536**** (2,342.003) | -7,043.068*** (2,384.335) | -66.942*** (25.149) | -56.320** (25.453) | 0.117*** (0.044) |
| BMI | -384.593**** (101.909) | -812.581**** (103.751) | -1.904* (1.094) | -2.038* (1.108) | 0.004** (0.002) |

| | | | | | |
|---|---|---|---|---|---|
| Mg x Age | -1.147$^{***}$ (0.406) | -1.016$^{**}$ (0.413) | | | |
| Constant | 293,482.300$^{****}$ (6,974.463) | 71,949.370$^{****}$ (7,100.527) | 2,614.836$^{****}$ (74.874) | 2,530.250$^{****}$ (75.777) | 2.789$^{****}$ (0.131) |
| Observations | 5,766 | 5,766 | 5,766 | 5,766 | 5,766 |

The significance levels were indicated as *p < 0.05, **p < 0.01, and ***p < 0.001. Abbreviations: SE, standard error; Mg, magnesium; GM, gray matter; WM, white matter; LHC, left hippocampal; RHC, right hippocampal; WMLs, white matter lesions; HDL, high-density lipoprotein; ; BMI, body mass index; ICV, intracranial volume.. The data is presented as unstandardized Beta correlation with the standard error (+/- SE), where Beta represents the effect size of a 1 mg increase in Mg intake, expressed in SD units of the dependent variable. It should be noted that the hierarchical analysis results of the association between Mg intake and brain volumes, including GM, WM, LHC, RHC, and WMLs. Model 1 was adjusted for the main covariates, including age, sex, education, and intracranial volume (ICV). Model 2 was additionally adjusted for other covariates, such as antihypertensive medication, high-density lipoprotein (HDL), cholesterol, diabetes mellitus, smoking status, higher education, physical activity, alcohol intake and BMI. Finally, Model 3 tested two-way interactions between Mg x Age, Mg x BP medication, and Mg x physical activity.

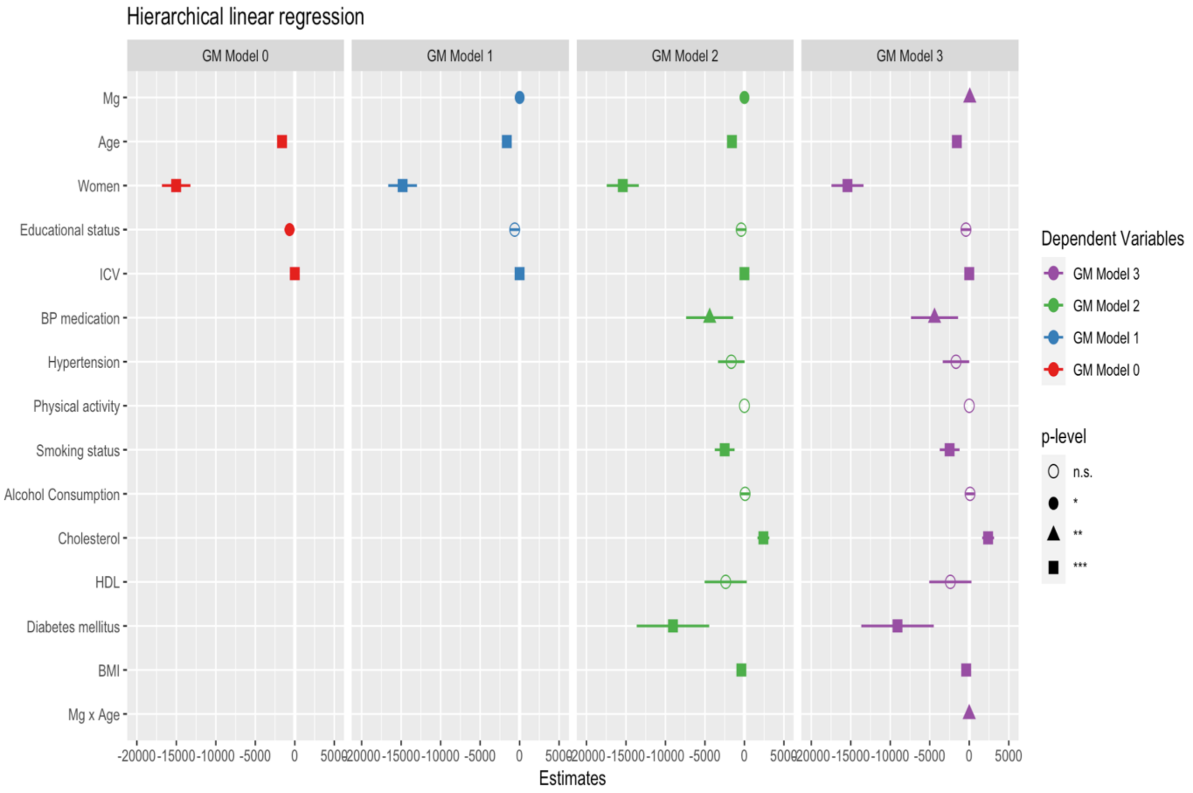


Figure S8. The figure presents the results of hierarchical regression analysis examining the association between dietary magnesium (Mg) intake and gray matter volume (GM) in the UK Biobank study. The data is presented as unstandardized Beta correlation with the standard error (+/- SE), where Beta represents the effect size of a 1 mg increase in Mg intake, expressed in SD units of the dependent variable. Model 1 was adjusted for the main covariates including age, sex, education, and intracranial volume (ICV). Model 2 was additionally adjusted for other covariates: antihypertensive medication, high-density lipoprotein (HDL) cholesterol, diabetes mellitus, smoking status, higher education, physical activity, alcohol intake, and body mass index (BMI). Model 3 additionally tested the two-way interactions between Mg x Age. Note: Error bars represent standard error from the same model. Statistical significance was denoted by *p<0.05, **p<0.01, and ***p<0.001.

Table S7. The association between dietary Mg intake and gray matter (GM) volume in the UK Biobank Study.

| | Gray matter volume | | | |
|---|---|---|---|---|
| | Model 0 | Model 1 | Model 2 | Model 3 |
| Mg | | 6.044** (3.082) | 6.705** (3.078) | 70.006*** (22.615) |
| Age | -1,616.359**** (51.282) | -1,621.210**** (51.329) | -1,579.770**** (53.072) | -1,567.023**** (53.231) |
| Women | -15,020.310**** (923.224) | -14,814.410**** (928.951) | -15,404.120**** (1,038.134) | -15,418.990**** (1,037.518) |
| Educational status | -663.541** (327.646) | -619.240* (328.344) | -410.947 (330.253) | -416.215 (330.058) |
| ICV | 0.303**** (0.003) | 0.303**** (0.003) | 0.304**** (0.003) | 0.304**** (0.003) |
| BP medication | | | -4,401.201*** (1,522.114) | -4,394.743*** (1,521.193) |
| Hypertension | | | -1,662.840* (857.717) | -1,678.662* (857.215) |
| Physical activity | | | -0.055 (0.167) | -0.047 (0.167) |
| Smoking status | | | -2,499.346**** (641.846) | -2,484.141**** (641.480) |
| Alcohol Consumption | | | 88.096 (290.463) | 103.314 (290.337) |
| Cholesterol | | | 2,402.037**** (384.596) | 2,389.700**** (384.387) |
| HDL | | | -2,372.179* (1,364.447) | -2,391.177* (1,363.636) |
| Diabetes mellitus | | | -9,050.092**** (2,343.399) | -9,080.536**** (2,342.003) |
| BMI | | | -385.419**** (101.971) | -384.593**** (101.909) |
| Mg x Age | | | | -1.147*** (0.406) |
| Constant | 295,807.500**** (6,021.507) | 296,184.200**** (6,023.089) | 293,952.700**** (6,976.707) | 293,482.300**** (6,974.463) |
| Observations | 5,776 | 5,776 | 5,766 | 5,766 |

Significance levels were indicated as *p < 0.05, **p < 0.01, and ***p < 0.001. Abbreviations: SE, standard error; Mg, magnesium; GM, gray matter; HDL, high-density lipoprotein; BMI, body mass index; ICV, intracranial volume. The data presented here shows unstandardized Beta correlation with the standard error (+/- SE), where Beta represents the effect size of a 1 mg increase in Mg intake, expressed in SD units of the dependent variable. Note that the hierarchical analysis results of the association between Mg intake and GM volume. Model 1 was adjusted for the main covariates including age, sex, education and ICV. Model 2 was additionally adjusted for

other covariates: antihypertensive medication, HDL, cholesterol, diabetes mellitus, smoking status, higher education, physical activity, alcohol intake and BMI. Model 3 was additionally tested for two-way interactions between Mg x Age.

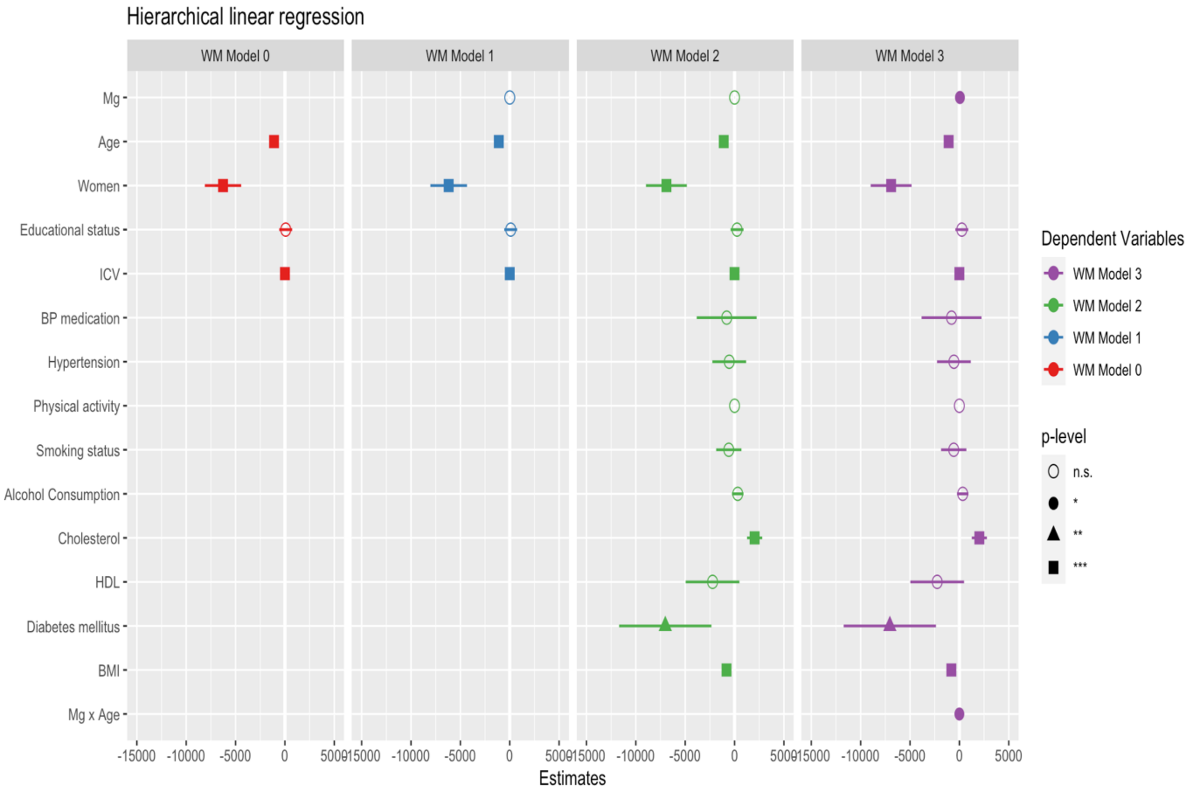


Figure S9. The figure presents the results of hierarchical regression analysis examining the association between dietary magnesium (Mg) intake and white matter volume (WM) in the UK Biobank study. The data is presented as unstandardized Beta correlation with the standard error (+/- SE), where Beta represents the effect size of a 1 mg increase in Mg intake, expressed in SD units of the dependent variable. Model 1 was adjusted for the main covariates including age, sex, education, and intracranial volume (ICV). Model 2 was additionally adjusted for other covariates: antihypertensive medication, high-density lipoprotein (HDL) cholesterol, diabetes mellitus, smoking status, higher education, physical activity, alcohol intake, and BMI. Model 3 additionally tested the two-way interactions between Mg x Age. Note that error bars represent the standard error from the same model. Statistical significance was denoted by *p<0.05, **p<0.01, and ***p<0.001.

Table S8. The association between dietary Magnesium intake and white matter (WM) volume in the UK Biobank Study.

| | White matter volume | | | |
|---|---|---|---|---|
| | Model 0 | Model 1 | Model 2 | Model 3 |
| Mg | | 2.528 (3.139) | 2.027 (3.133) | 58.106** (23.024) |
| Age | -1,103.950**** (52.200) | -1,105.979**** (52.263) | -1,095.880**** (54.022) | -1,084.587**** (54.193) |
| Women | -6,272.011**** (939.753) | -6,185.903**** (945.844) | -6,901.047**** (1,056.720) | -6,914.226**** (1,056.271) |
| Educational status | 80.900 (333.512) | 99.426 (334.315) | 252.689 (336.166) | 248.022 (336.024) |
| ICV | 0.310**** (0.003) | 0.309**** (0.003) | 0.312**** (0.003) | 0.311**** (0.003) |
| BP medication | | | -797.377 (1,549.366) | -791.655 (1,548.689) |
| Hypertension | | | -539.271 (873.073) | -553.288 (872.709) |
| Physical activity | | | 0.306* (0.170) | 0.313* (0.170) |
| Smoking status | | | -585.591 (653.338) | -572.120 (653.075) |
| Alcohol Consumption | | | 324.330 (295.664) | 337.812 (295.585) |
| Cholesterol | | | 2,033.532**** (391.481) | 2,022.601**** (391.335) |
| HDL | | | -2,228.239 (1,388.876) | -2,245.070 (1,388.284) |
| Diabetes mellitus | | | -7,016.096*** (2,385.355) | -7,043.068*** (2,384.335) |
| BMI | | | -813.312**** (103.796) | -812.581**** (103.751) |
| Mg x Age | | | | -1.016** (0.413) |
| Constant | 63,177.360**** (6,129.309) | 63,334.900**** (6,132.617) | 72,366.060**** (7,101.617) | 71,949.370**** (7,100.527) |
| Observations | 5,776 | 5,776 | 5,766 | 5,766 |
| *Note:* | .* p<0.05; ** p<0.01; *** p<0.001 | | | |

Significance levels were indicated as *p < 0.05, **p < 0.01, and ***p < 0.001. Abbreviations: SE, standard error; Mg, magnesium; WM, white matter; HDL, high-density lipoprotein; ; BMI, body mass index; ICV, intracranial volume. The data presented here shows unstandardized Beta correlation with the standard error (+/- SE), where Beta represents the effect size of a 1 mg increase in Mg intake, expressed in SD units of the dependent variable. Note that the hierarchical analysis results of the association between Mg intake and WM volume. Model 1 was

adjusted for the main covariates including age, sex, education, and ICV. Model 2 was additionally adjusted for other covariates: antihypertensive medication, HDL, cholesterol, diabetes mellitus, smoking status, higher education, physical activity, alcohol intake and BMI. Model 3 was additionally tested for two-way interactions between Mg x Age.

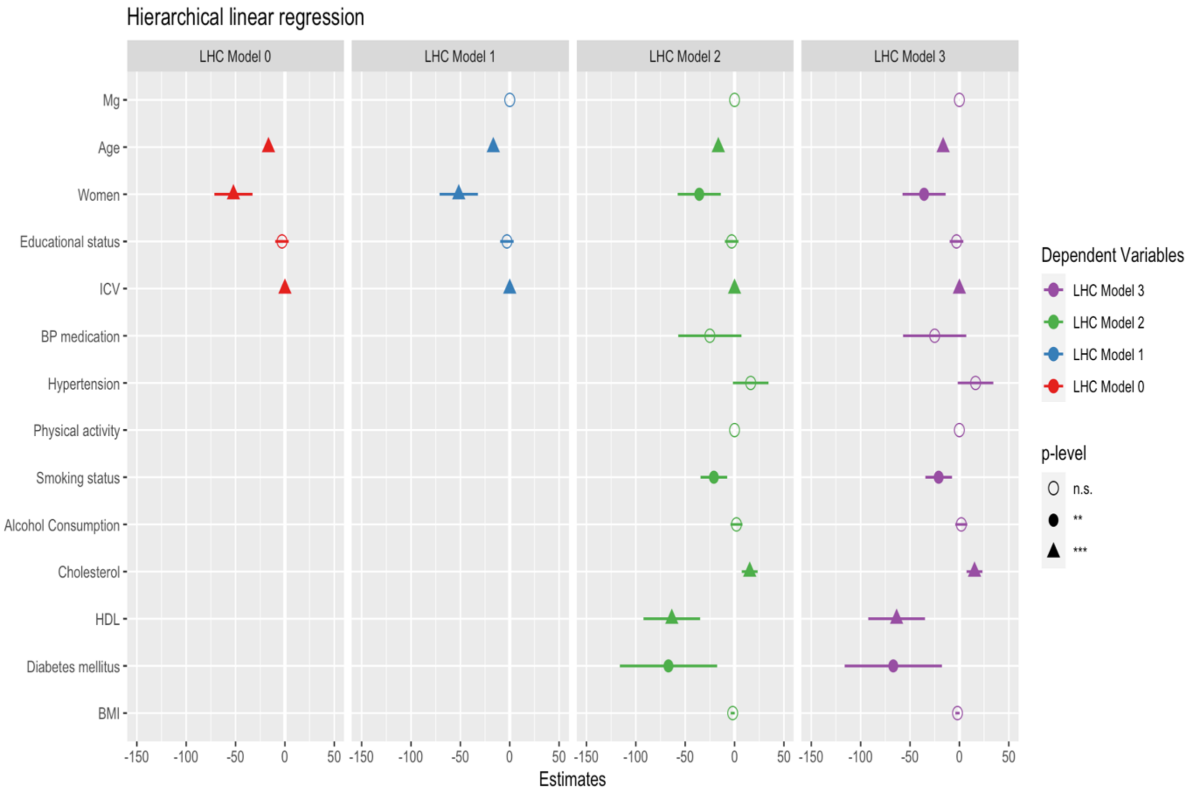


Figure S10. The figure presents the results of hierarchical regression analysis examining the association between magnesium (Mg) intake and left hippocampus (LHC) volume at the UK Biobank study. The data is presented as unstandardized Beta correlation with the standard error (+/- SE), where Beta represents the effect size of a 1 mg increase in Mg intake, expressed in SD units of the dependent variable. Model 1 was adjusted for the main covariates including age, sex, education, and intracranial volume (ICV). Model 2 was additionally adjusted for other covariates: antihypertensive medication, high-density lipoprotein (HDL) cholesterol, diabetes mellitus, smoking status, higher education, physical activity, alcohol intake, and body mass index (BMI). Model 3 was additionally tested for two-way interactions between covariates. Note that error bars represent standard errors from the same model. Significance is denoted by * $p<0.05$, ** $p<0.01$, and *** $p<0.001$.

Table S9. The association between dietary Mg intake and left hippocampus (LHC) volume in the UK Biobank Study.

| | Left hippocampal volume | | | |
|---|---|---|---|---|
| | Model 0 | Model 1 | Model 2 | Model 3 |
| Mg | | 0.015 (0.033) | 0.020 (0.033) | 0.020 (0.033) |
| Age | -16.658$^{****}$ (0.548) | -16.670$^{****}$ (0.548) | -16.450$^{****}$ (0.570) | -16.450$^{****}$ (0.570) |
| Women | -52.155$^{****}$ (9.860) | -51.647$^{****}$ (9.924) | -35.749$^{***}$ (11.141) | -35.749$^{***}$ (11.141) |
| Educational status | -2.966 (3.499) | -2.857 (3.508) | -2.913 (3.544) | -2.913 (3.544) |
| ICV | 0.001$^{****}$ (0.00003) | 0.001$^{****}$ (0.00003) | 0.001$^{****}$ (0.00003) | 0.001$^{****}$ (0.00003) |
| BP medication | | | -25.032 (16.335) | -25.032 (16.335) |
| Hypertension | | | 16.368$^{*}$ (9.205) | 16.368$^{*}$ (9.205) |
| Physical activity | | | -0.001 (0.002) | -0.001 (0.002) |
| Smoking status | | | -20.957$^{***}$ (6.888) | -20.957$^{***}$ (6.888) |
| Alcohol Consumption | | | 1.949 (3.117) | 1.949 (3.117) |
| Cholesterol | | | 15.351$^{****}$ (4.127) | 15.351$^{****}$ (4.127) |
| HDL | | | -63.564$^{****}$ (14.643) | -63.564$^{****}$ (14.643) |
| Diabetes mellitus | | | -66.942$^{***}$ (25.149) | -66.942$^{***}$ (25.149) |
| BMI | | | -1.904$^{*}$ (1.094) | -1.904$^{*}$ (1.094) |
| Constant | 2,594.188$^{****}$ (64.310) | 2,595.118$^{****}$ (64.348) | 2,614.836$^{****}$ (74.874) | 2,614.836$^{****}$ (74.874) |
| Observations | 5,776 | 5,776 | 5,766 | 5,766 |

Significance levels were indicated as *p < 0.05, **p < 0.01, and ***p < 0.001. Abbreviations: SE, standard error; Mg, magnesium; LHC, left hippocampus volume; HDL, high-density lipoprotein; ; BMI, body mass index; ICV, intracranial volume. The data presented here shows unstandardized Beta correlation with the standard error (+/-SE), where Beta represents the effect size of a 1 mg increase in Mg intake, expressed in SD units of the dependent variable. Note that the hierarchical analysis results of the association between Mg intake and LHC volume. Model 1 was adjusted for the main covariates including age, sex, education, and ICV. Model 2 was additionally adjusted for other covariates: antihypertensive medication, HDL, cholesterol, diabetes mellitus, smoking status, higher education, physical activity, alcohol intake and BMI. Model 3 was additionally tested the two-way interactions between Mg x covariates.

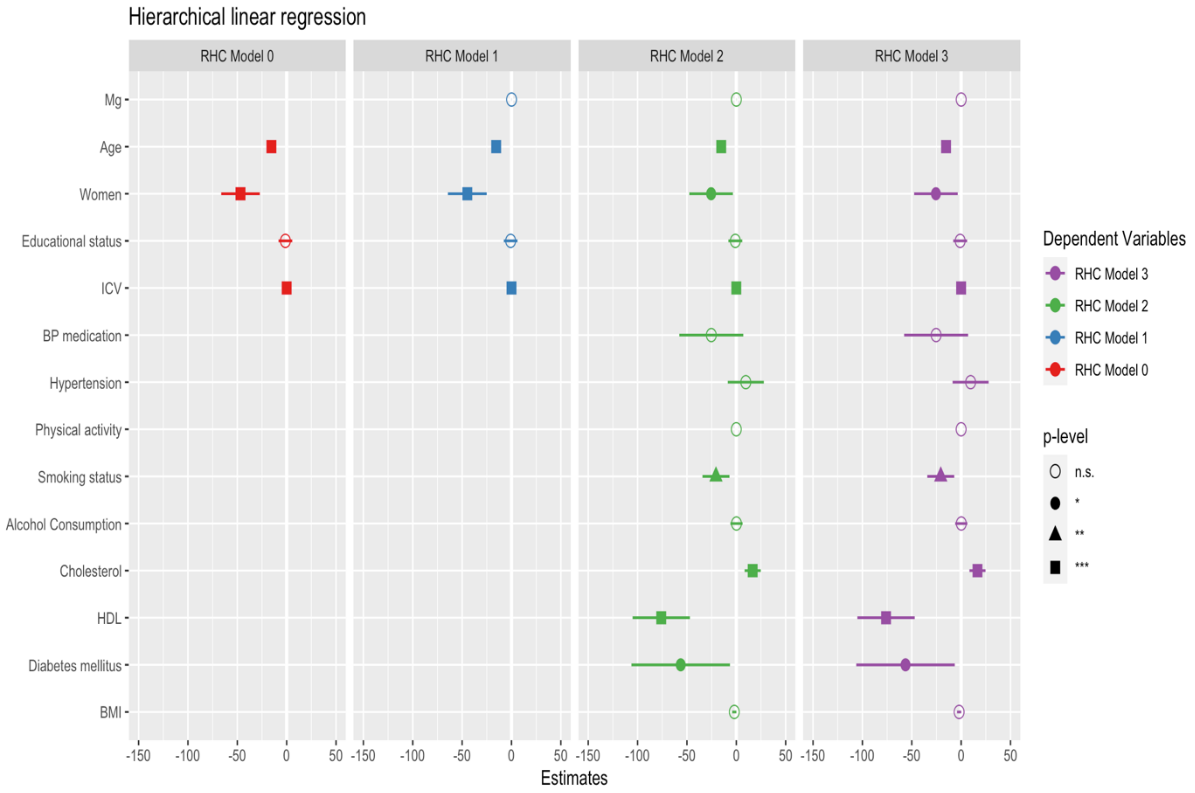


Figure S11. The figure presents the results of hierarchical regression analysis examining the association between Magnesium intake and right hippocampal (RHC) volume in the UK Biobank study. The data are presented as unstandardized Beta correlation with the standard error (+/- SE), where Beta represents the effect size of a 1 mg increase in Mg intake, expressed in SD units of the dependent variable. Model 1 was adjusted for the main covariates, including age, sex, education, and ICV. Model 2 was additionally adjusted for other covariates: antihypertensive medication, high-density lipoprotein (HDL) cholesterol, diabetes mellitus, smoking status, higher education, physical activity, alcohol intake, and BMI. Model 3 additionally tested the two-way interactions between Mg x covariates. Note. Error bars represent standard error from the same model. Significance is at * $p<0.05$; ** $p<0.01$; *** $p<0.001$.

Table S10. The association between dietary Mg intake and right hippocampal (RHC) volume in the UK Biobank Study.

| | Right hippocampal volume | | | |
|---|---|---|---|---|
| | Model 0 | Model 1 | Model 2 | Model 3 |
| Mg | | $0.059^{*}$ (0.033) | $0.062^{*}$ (0.033) | $0.062^{*}$ (0.033) |
| Age | $-15.472^{****}$ (0.554) | $-15.519^{****}$ (0.554) | $-15.261^{****}$ (0.576) | $-15.261^{****}$ (0.576) |
| Women | $-46.760^{****}$ (9.971) | $-44.754^{****}$ (10.034) | $-25.528^{**}$ (11.276) | $-25.528^{**}$ (11.276) |
| Educational status | -1.294 (3.539) | -0.862 (3.546) | -0.976 (3.587) | -0.976 (3.587) |
| ICV | $0.001^{****}$ (0.00003) | $0.001^{****}$ (0.00003) | $0.001^{****}$ (0.00003) | $0.001^{****}$ (0.00003) |
| BP medication | | | -25.334 (16.532) | -25.334 (16.532) |
| Hypertension | | | 9.619 (9.316) | 9.619 (9.316) |
| Physical activity | | | 0.001 (0.002) | 0.001 (0.002) |
| Smoking status | | | $-20.611^{***}$ (6.971) | $-20.611^{***}$ (6.971) |
| Alcohol Consumption | | | 0.170 (3.155) | 0.170 (3.155) |
| Cholesterol | | | $16.573^{****}$ (4.177) | $16.573^{****}$ (4.177) |
| HDL | | | $-76.043^{****}$ (14.820) | $-76.043^{****}$ (14.820) |
| Diabetes mellitus | | | $-56.320^{**}$ (25.453) | $-56.320^{**}$ (25.453) |
| BMI | | | $-2.038^{*}$ (1.108) | $-2.038^{*}$ (1.108) |
| Constant | $2,496.191^{****}$ (65.034) | $2,499.862^{****}$ (65.056) | $2,530.250^{****}$ (75.777) | $2,530.250^{****}$ (75.777) |
| Observations | 5,776 | 5,776 | 5,766 | 5,766 |

Significance levels were indicated as *p < 0.05, **p < 0.01, and ***p < 0.001. Abbreviations: SE, standard error; Mg, magnesium; LHC, right hippocampus volume HDL, high-density lipoprotein; BMI, body mass index; ICV, intracranial volume. The data presented here shows unstandardized Beta correlation with the standard error (+/-SE), where Beta represents the effect size of a 1 mg increase in Mg intake, expressed in SD units of the dependent variable. Note that the hierarchical analysis results of the association between Mg intake and RHC volume. Model 1 was adjusted for the main covariates including age, sex, education, and ICV. Model 2 was additionally adjusted for other covariates: antihypertensive medication, HDL, cholesterol, diabetes mellitus, smoking status, higher education, physical activity, alcohol intake and BMI. Model 3 was additionally tested the two-way interactions between Mg x covariates.

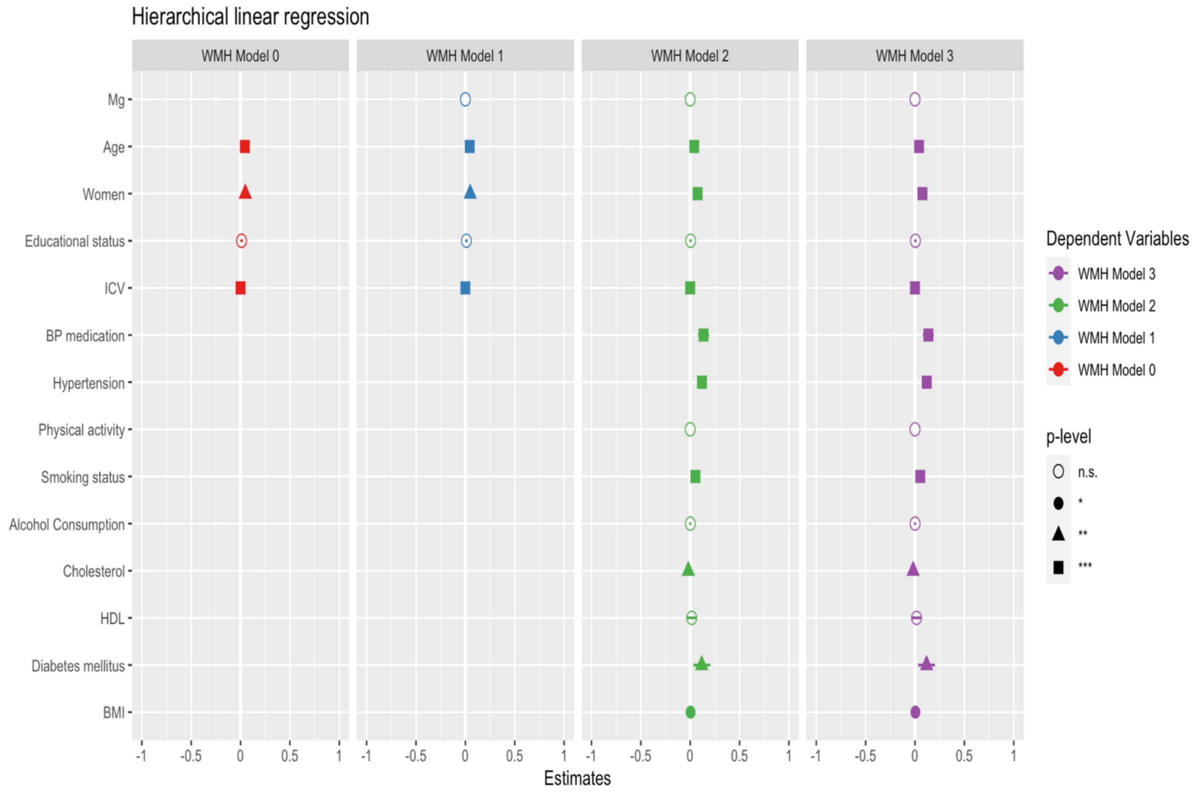


Figure S12. Hierarchical regression analysis results of the association between Magnesium intake and white matter lesions (WMLs) at the UK Biobank study. The data is presented as unstandardized Beta correlation with the standard error (+/- SE), where Beta represents the effect size of a 1 mg increase in Mg intake, expressed in SD units of the dependent variable. Model 1 was adjusted for the main covariates including age, sex, education, and ICV. Model 2 was additionally adjusted for other covariates: antihypertensive medication, high-density lipoprotein (HDL), cholesterol, diabetes mellitus, smoking status, higher education, physical activity, alcohol intake, and BMI. Model 3 was additionally tested for the two-way interactions between Mg and covariates. Note. Error bars represent standard error from the same model. Significance is denoted by *p<0.05, **p<0.01, and ***p<0.001.

Table S11. The association between dietary Mg intake and white matter lesions (WMLs) in the UK Biobank Study.

| | White matter lesions | | | |
|---|---|---|---|---|
| | Model 0 | Model 1 | Model 2 | Model 3 |
| Mg | | 0.00004 (0.0001) | 0.00003 (0.0001) | 0.00003 (0.0001) |
| Age | 0.044**** (0.001) | 0.044**** (0.001) | 0.041**** (0.001) | 0.041**** (0.001) |
| Women | 0.048*** (0.017) | 0.049*** (0.017) | 0.075**** (0.019) | 0.075**** (0.019) |
| Educational status | 0.010* (0.006) | 0.010* (0.006) | 0.004 (0.006) | 0.004 (0.006) |
| ICV | 0.00000**** (0.00000) | 0.00000**** (0.00000) | 0.00000**** (0.00000) | 0.00000**** (0.00000) |
| BP medication | | | 0.135**** (0.029) | 0.135**** (0.029) |
| Hypertension | | | 0.119**** (0.016) | 0.119**** (0.016) |
| Physical activity | | | 0.00000 (0.00000) | 0.00000 (0.00000) |
| Smoking status | | | 0.053**** (0.012) | 0.053**** (0.012) |
| Alcohol Consumption | | | 0.001 (0.005) | 0.001 (0.005) |
| Cholesterol | | | -0.019*** (0.007) | -0.019*** (0.007) |
| HDL | | | 0.016 (0.026) | 0.016 (0.026) |
| Diabetes mellitus | | | 0.117*** (0.044) | 0.117*** (0.044) |
| BMI | | | 0.004** (0.002) | 0.004** (0.002) |
| Constant | 2.782**** (0.113) | 2.784**** (0.113) | 2.789**** (0.131) | 2.789**** (0.131) |
| Observations | 5,776 | 5,776 | 5,766 | 5,766 |

Significance levels were indicated as *p < 0.05, **p < 0.01, and ***p < 0.001. Abbreviations: SE, standard error; Mg, magnesium; WMLs, white matter lesions; HDL, high-density lipoprotein; BMI, body mass index; ICV, intracranial volume. The data presented here shows unstandardized Beta correlation with the standard error (+/- SE), where Beta represents the effect size of a 1 mg increase in Mg intake, expressed in SD units of the dependent variable. Note that the hierarchical analysis results of the association between Mg intake and WMLs volume. Model 1 was adjusted for the main covariates including age, sex, education, and ICV. Model 2 was additionally adjusted for other covariates: antihypertensive medication, HDL, cholesterol, diabetes mellitus, smoking status, higher education, physical activity, alcohol intake and BMI. Model 3 was additionally tested the two-way interactions between Mg x covariates.

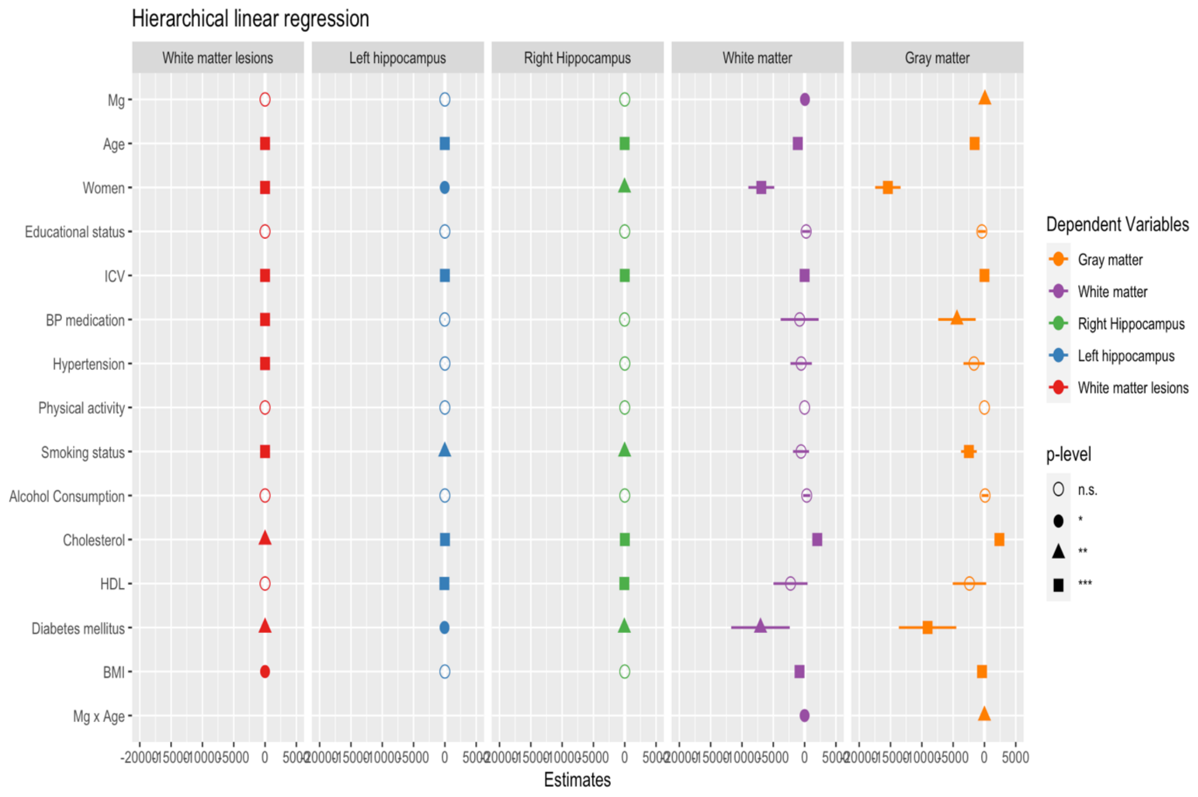


Figure S13. The figure presents the results of hierarchical regression analysis examining the association between magnesium intake and brain volumes, including gray matter (GM), white matter (WM), left hippocampal (LHC), right hippocampal (RHC), and white matter lesions (WMLs), at the UK Biobank study. The data is presented as unstandardized Beta correlation with the standard error (+/- SE), where Beta represents the effect size of a 1 mg increase in Mg intake, expressed in SD units of the dependent variable. Model 1 was adjusted for the main covariates, including age, sex, education, and intercranial volume (ICV). Model 2 was additionally adjusted for other covariates, including antihypertensive medication, high-density lipoprotein (HDL), cholesterol, diabetes mellitus, smoking status, higher education, physical activity, alcohol intake, and body mass index (BMI). Model 3 was additionally tested for the two-way interactions between Mg and Age. Note. Error bars represent standard error from the same model. Significance is denoted by *p<0.05, **p<0.01, and ***p<0.001.

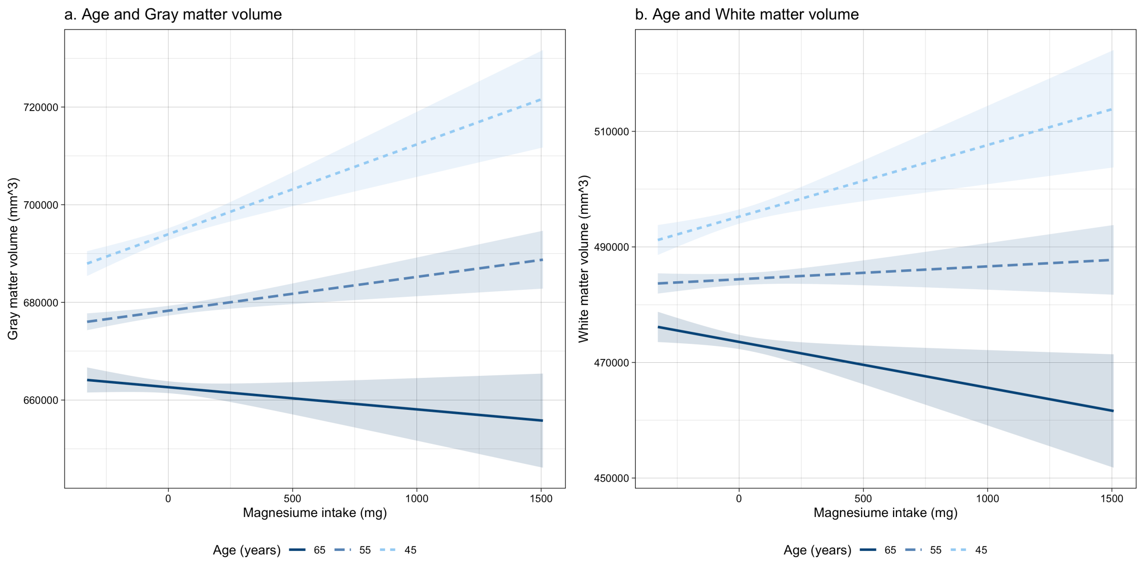


Figure S14. The interaction effects between dietary magnesium intake and age in predicting a) gray matter volume and b) white matter volume are shown. Note. The shadow represents the 95% confidence intervals (CI).

Table S12. Association between dietary Mg intake and inflammatory markers controlled for calcium (Ca) and Energy intake.

| | Leukocytes ($10^9$ cells/L) | ESR ($10^{12}$ cells/L) | hs-CRP Level (mg/L) | GlycA (mmol/L) |
|---|---|---|---|---|
| | Beta (CI) | Beta (CI) | Beta (CI) | Beta (CI) |
| Dietary Mg intake | -0.0002*** (-0.0003, -0.0001) | -0.0001* (-0.0002, 0.00001) | -0.001*** (-0.002, -0.001) | -0.0003*** (-0.001, -0.0001) |
| Energy intake | 0.00000** (0.00000, 0.00001) | 0.00000 (-0.00000, 0.00001) | 0.00002*** (0.00001, 0.00004) | -0.00000 (-0.00000, 0.00000) |
| Calcium | 0.00002** (0.00000, 0.00004) | 0.00002 (-0.00001, 0.00005) | 0.0001*** (0.00003, 0.0002) | -0.00000 (-0.00002, 0.00001) |
| Constant | -10.287*** (-10.366, -10.208) | 4.529*** (4.421, 4.637) | -2.909*** (-3.224, -2.595) | 0.630*** (0.525, 0.736) |
| Observations | 5,766 | 5,766 | 5,468 | 1,417 |

Significance. * p<0.05; ** p<0.01; *** p<0.001. Abbreviations: CI-confidence interval - standard error; Mg - magnesium; hs-CRP - high-sensitivity C-reactive protein; GlycA - Glycoprotein acetylation. Note: Sensitivity analysis controlled for the calcium levels and energy intake in the association between dietary Mg intake and inflammatory markers, including leukocytes, erythrocytes, hs-CRP, and GlycA, using data from the UK Biobank study. The data represents unstandardized beta coefficients with 95% Confidence Interval. Beta values correspond to a 1mg unit increment in Mg intake variables.

Table 13 Association between dietary Mg intake and inflammatory markers in men and women.

| | Leukocytes ($10^9$ cells/L) | | ESR ($10^{12}$ cells/L) | | hs-CRP Level (mg/L) | | GlycA (mmol/L) | |
|---|---|---|---|---|---|---|---|---|
| | Men | Women | Men | Women | Men | Women | Men | Women |
| | | Beta (CI) | Beta (CI) | Beta (CI) | Beta (CI) | Beta (CI) | Beta (CI) | Beta (CI) |
| Mg | -0.00003 (-0.0001, 0.0001) | -0.0001* (-0.0002, 0.00001) | -0.00004 (-0.0001, 0.0001) | 0.00004 (-0.0001, 0.0001) | -0.001*** (-0.001, -0.0003) | -0.001*** (-0.001, -0.0004) | -0.0004*** (-0.001, -0.0001) | -0.0002 (-0.001, 0.0001) |
| Constant | -4.418*** (-4.541, -4.296) | -4.137*** (-4.242, -4.031) | 4.860*** (4.681, 5.038) | 3.903*** (3.767, 4.039) | -1.914*** (-2.402, -1.426) | -3.178*** (-3.599, -2.757) | 0.747*** (0.585, 0.909) | 0.546*** (0.397, 0.695) |
| Observations | 2,744 | 3,022 | 2,744 | 3,022 | 2,598 | 2,870 | 663 | 754 |

Significance. * p<0.05; ** p<0.01; *** p<0.001. Abbreviations: CI-confidence interval - standard error; Mg - magnesium; hs-CRP - high-sensitivity C-reactive protein; GlycA - Glycoprotein acetylation. Note: The sensitivity analysis stratified the sample by gender to investigate the association between dietary Mg intake and inflammatory markers, including leukocytes, erythrocytes, hs-CRP, and GlycA, using data from the UK Biobank study. The data represents unstandardized beta coefficients with 95% Confidence Interval. Beta values correspond to a 1mg unit increment in Mg intake variables.

Table 14. Association between dietary Mg intake and brain volumes and WMLs in men and women.

| | Gray matter volume ($mm^3$) | | White matter volume ($mm^3$) | | Left hippocampal volume ($mm^3$) | | Right hippocampal volume ($mm^3$) | | White matter lesions ($mm^3$) | |
|---|---|---|---|---|---|---|---|---|---|---|
| | Men | Women | Men | Women | Men | Women | Men | Women | Men | Women |
| | Beta (CI) | Beta (CI) | Beta (CI) | Beta (CI) | Beta (CI) | Beta (CI) | Beta (CI) | Beta (CI) | Beta (CI) | Beta (CI) |
| Mg | 92.679*** (27.156, 158.203) | -16.703 (-78.089, 44.683) | 20.693 (-46.143, 87.529) | 19.363 (-42.919, 81.645) | -0.005 (-0.098, 0.088) | 0.045 (-0.046, 0.135) | 0.058 (-0.037, 0.153) | 0.064 (-0.027, 0.154) | 0.00001 (-0.0001, 0.0002) | 0.0001 (-0.0001, 0.0002) |
| Constant | 303,142.800*** (270,350.600, 335,935.000) | 247,172.600*** (220,318.200, 274,027.100) | 100,033.700*** (66,584.530, 133,482.800) | 33,144.460** (5,898.086, 60,390.840) | 2,797.957*** (2,567.387, 3,028.528) | 2,395.687*** (2,209.628, 2,581.745) | 2,755.521*** (2,519.826, 2,991.217) | 2,263.706*** (2,077.731, 2,449.682) | 2.589*** (2.209, 2.969) | 2.987*** (2.641, 3.332) |
| Observation | 2,744 | 3,022 | 2,744 | 3,022 | 2,744 | 3,022 | 2,744 | 3,022 | 2,744 | 3,022 |

Significance. * $p<0.05$; ** $p<0.01$; *** $p<0.001$. Abbreviations: CI - confidence interval; Mg - magnesium; GM - grey matter; The sensitivity analysis stratified the sample by gender to investigate the association between dietary Mg intake and brain volumes, including GM, WM, LHC, RHC, and WMLs. The data represents unstandardized beta coefficients with 95% Confidence Interval. Beta values correspond to a 1mg unit increment in Mg intake variables.
